# GABAergic Progenitor Cell Graft Rescues Cognitive Deficits in Fragile X Syndrome Mice

**DOI:** 10.1002/advs.202411972

**Published:** 2025-01-17

**Authors:** Chen Wang, Jia‐Yu Liu, Li‐Da Su, Xin‐Tai Wang, Yu‐Peng Bian, Zhao‐Xiang Wang, Lu‐Yu Ye, Xin‐Jiang Lu, Lin Zhou, Wei Chen, Wei Yang, Jun Liu, Luxi Wang, Ying Shen

**Affiliations:** ^1^ Department of Neurology Institute of Neuroscience Key Laboratory of Neurogenetics and Channelopathies of Guangdong Province and the Ministry of Education of China The Second Affiliated Hospital Guangzhou Medical University Guangzhou 510260 China; ^2^ Department of Physiology and Department of Psychiatry Sir Run Run Shaw Hospital Zhejiang University School of Medicine Hangzhou 310058 China; ^3^ Zhejiang Development & Planning Institute Hangzhou 310030 China; ^4^ Neuroscience Care Unit Key Laboratory of Multiple Organ Failure of Ministry of Education the Second Affiliated Hospital of Zhejiang University School of Medicine Hangzhou 310009 China; ^5^ Key Laboratory of the Diagnosis and Treatment of Severe Trauma and Burn of Zhejiang Province Hangzhou 310009 China; ^6^ Institute of Life Sciences College of Life and Environmental Sciences Hangzhou Normal University Hangzhou 311121 China; ^7^ Center for Brain Health the Fourth Affiliated Hospital of School of Medicine and International School of Medicine International Institutes of Medicine Zhejiang University Yiwu 322000 China; ^8^ Zhejiang Lab Hangzhou 311500 China; ^9^ Department of Biophysics Zhejiang University School of Medicine Hangzhou 310058 China; ^10^ Department of Physiology Zhejiang University School of Medicine Hangzhou 310058 China; ^11^ Key Laboratory for Precision Diagnosis Treatment and Clinical Translation of Rare Diseases of Zhejiang Province Zhejiang University School of Medicine Hangzhou 310058 China

**Keywords:** brain oscillation, cell therapy, hippocampus, long‐term potentiation, medial ganglionic eminence

## Abstract

Fragile X syndrome (FXS) is an inherited neurodevelopmental disorder characterized by a range of clinical manifestations with no effective treatment strategy to date. Here, transplantation of GABAergic precursor cells from the medial ganglionic eminence (MGE) is demonstrated to significantly improve cognitive performance in Fmr1 knockout (KO) mice. Within the hippocampus of Fmr1‐KO mice, MGE‐derived cells from wild‐type donor mice survive, migrate, differentiate into functionally mature interneurons, and form inhibitory synaptic connections with host pyramidal neurons. MGE cell transplantation restores Ras‐PKB signaling in pyramidal neurons, enhances AMPA receptor trafficking, rescues synaptic plasticity, and corrects abnormal hippocampal neural oscillations. These findings highlight the potential of GABAergic precursor cell transplantation as a promising therapeutic strategy for FXS.

## Graphical abstract

In adult Fmr1 knockout mice, the engrafted GABAergic progenitor cells from the medial ganglionic eminence can differentiate into interneurons and restore cognitive deficits by activating Ras‐PI3K signaling, promoting synaptic receptor trafficking and long‐term potentiation, as well as restoring circuit oscillations. These findings highlight the clinical significance of transplanting GABAergic progenitor cells for the treatment of Fragile X syndrome.

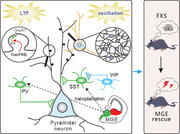

## 1 Introduction

Fragile X syndrome (FXS) is an inherited neurodevelopmental disorder characterized by a spectrum of clinical manifestations, including cognitive impairments.^[^ ^1^ ^]^ The absence of the fragile X mental retardation protein (FMRP) caused by mutations in the Fmr1 gene has been recognized as a causative factor for FXS.^[^ ^2^ ^]^ Consequently, Fmr1‐KO mice are widely used as an animal model for investigating the pathophysiological mechanisms underlying FXS.^[^ ^3^ ^]^ Emerging evidence indicates that aberrant expression of synaptic receptors, disrupted synaptic transmission, and impaired synaptic protein signaling are central to the etiology of FXS.^[^ ^4^, ^5^, ^6^, ^7^, ^8^, ^9^ ^]^ Notably, type I metabolic glutamate receptors (mGluRs) are closely linked to FMRP: On one hand, mGluR5 activation or enhancement of mGluR‐mediated synaptic plasticity regulates FMRP synthesis, dephosphorylation, ubiquitination, and degradation;^[^ ^10^ ^]^ On the other hand, the absence of FMRP exacerbates mGluR‐induced long‐term depression (LTD), reducing synaptic connections, and potentially contributing to the cognitive deficits observed in FXS.^[^ ^11^, ^12^ ^]^ Consequently, preclinical studies and clinical trials have focused on mGluR agonists and inhibitors as potential therapeutic. Unfortunately, these efforts have not yet yielded successful outcomes, leaving effective treatment options for FXS elusive.^[^ ^13^ ^]^

Cell therapy has emerged as a promising approach for treating various human diseases. More than 200 clinical studies are employing stem cells to treat neurological diseases (Clinicaltrials.gov), the majority of which are multiple sclerosis, stroke, Parkinson's disease, and spinal cord injury. In particular, neural progenitor cells are particularly well‐suited for addressing biological defects in the brain.^[^ ^14^ ^]^ Notably, GABAergic progenitor cells, a specific subset of progenitor cells, have demonstrated the ability to migrate over long distances and functionally integrate into neural circuits without inducing tumors, teratomas, or immune responses.^[^ ^15^ ^]^ Moreover, GABAergic progenitor cells derived from the medial ganglionic eminence (MGE) have been shown to survive in the recipient brain and differentiate into mature GABAergic neurons.^[^ ^16^, ^17^, ^18^, ^19^ ^]^ However, whether GABAergic progenitor cell transplantation could serve as a viable therapeutic strategy for adult FXS, a psychiatric condition with a distinct pathogenic mechanism compared to neurodegenerative diseases, remains unexplored.

Research has shown that FMRP plays a critical role in the development of interneurons (INs), with its deficiency leading to a widespread downregulation of GABAergic transmission.^[^ ^20^, ^21^ ^]^ Restoration of GABA receptors (GABARs) expression can modulate the excitability of pyramidal neurons (PNs) and improve cognitive behaviors in FXS mice.^[^ ^22^, ^23^ ^]^ These findings underscore the pivotal role of GABAergic transmission in the pathophysiology of FXS and offer a theoretical foundation for exploring the therapeutic potential of MGE‐derived GABAergic progenitor cells in FXS treatment.

In this study, we transplanted MGE cells derived from wild‐type (WT) embryonic mice into the hippocampi of adult Fmr1‐KO mice. Our findings demonstrate that the transplanted MGE cells successfully migrated within the hippocampus, differentiated into INs, and ameliorated cognitive deficits in Fmr1‐KO mice. The engrafted cells induced both cellular and circuitry improvements, including the restoration of Ras‐protein kinase B (PKB) signaling activity, recovery of long‐term potentiation (LTP), and correction of impaired hippocampal neural oscillations in Fmr1‐KO mice. These findings provide compelling evidence supporting MGE cell transplantation as a promising therapeutic strategy for FXS.

## 2 Results

### 2.1 Engrafted MGE Cells Migrate into Adult Hippocampus of Fmr1‐KO Mice and Express Markers of Inhibitory Neurons

**Figure 1:**
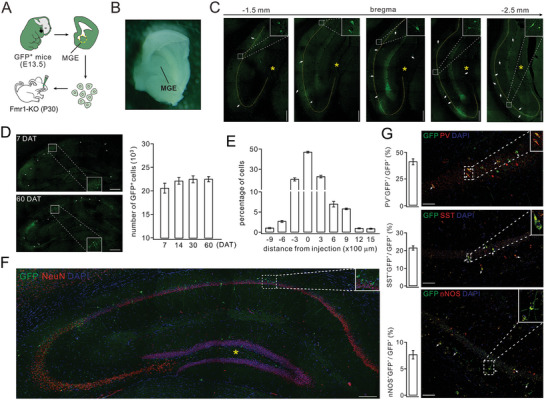
Transplanted MGE cells migrate to CA1 and express inhibitory neuronal markers in Fmr1‐KO mice. A) Schematic illustration depicting the extraction of MGE cells from embryonic GFP^+^ mice and their subsequent transplantation into Fmr1‐KO mice (P25‐30). B) An image presenting the dissection of MGE. C) Example images showing GFP^+^ MGE cells at 7 DAT (white arrowheads). Example MGE cells are shown in the insets. Yellow asterisk: injection site. Yellow dashed lines demarcate the CA3‐CA1 area. Scale bars, 500 µm. D) Left: example images showing GFP^+^ cells at 7 DAT and 60 DAT. Scale bars, 500 µm. The insets show example MGE cells. Right: the bar graphs present the quantification of GFP^+^ cells at different DATs. E) Distribution of transplanted MGE cells at 60 DAT. F) Example images showing labeling of NeuN and transplanted MGE cells at 60 DAT. Transplanted neurons dispersed upon grafting into recipient mice. The magnification shows example NeuN^+^GFP^+^ differentiated cells. Scale bar, 500 µm. G) Example images showing GFP^+^ cells (60 DAT) co‐expressing PV, SST, or nNOS. Arrowheads annotate co‐labeled cells. The magnifications show examples of PV^+^GFP^+^ (upper), SST^+^GFP^+^ (middle), and nNOS^+^GFP^+^ (lower) differentiated cells. Scale bars, 50 µm. Bar graphs indicate the proportions of PV^+^ (41.3% ± 2.8%), SST^+^ (21.6% ± 1.2%), and nNOS^+^ (7.7% ± 0.8%) cells among GFP^+^ MGE cells. *n*  = 4 per group. See Table S1 (Supporting Information) for all statistics, including *n* values, *p* values, and statistical tests.

The MGE of embryonic mice harbors a significant population of GABAergic progenitor cells characterized by the expressions of 2 transcription factors: forkhead box G1 (FOXG1) and NK2 homeobox 1 (NKX2.1).^[^ ^24^, ^25^ ^]^ In this study, MGE cells were isolated from GFP‐expressing (GFP^+^) WT donor mice and transplanted into the hippocampus of Fmr1‐KO mice at postnatal days 25–30 (**Figure** **1**A,B). Immunofluorescent staining of FOXG1 and NKX2.1 on primary cultures of engrafted MGE cells affirmed their origin to be from MGE rather than caudal ganglionic eminence (CGE) or lateral ganglionic eminence (LGE) (Figure S1, Supporting Information).

It is widely recognized that the mouse hippocampus, which is closely related to cognition, contains neural stem cells (NSCs) in the dentate gyrus (DG) region during adult stages.^[^ ^26^ ^]^ Given this supportive microenvironment, we selected the DG as the transplantation site. A total of ≈6 × 10^4^ MGE cells were bilaterally injected into the DG of Fmr1‐KO mice at P30. Over 7–60 days after transplantation (DAT), robust survival and migration of MGE cells were observed within the hippocampi of Fmr1‐KO mice (Figure 1C; Figure S2, Supporting Information). Notably on 7 DAT, GFP^+^ MGE cells had dispersed throughout the hippocampal subfields, including the distal CA1 area (Figure 1C), which receives efferent inputs from the lateral entorhinal cortex (LEC) via the temporoammonic (TA) path.^[^ ^27^ ^]^ Quantification of GFP^+^ cells revealed a survival rate of ≈35% at 7 DAT (*n* = 15), consistent with previous studies.^[^ ^19^ ^]^ Temporal analysis of MGE grafts was conducted by quantifying the number of GFP^+^ cells in the Fmr1‐KO hippocampi at DAT7, DAT14, DAT30, and DAT60. The results revealed that the numbers of GFP^+^ cells were 20.5 ± 0.8 × 10^3^ (*n* = 5; DAT7), 21.5 ± 0.7 × 10^3^ (*n* = 5; DAT14), 22.5 ± 0.6 × 10^3^ (*n* = 5; DAT30), and 22.9 ± 0.6 × 10^3^ (*n* = 5; DAT60) (Figure 1D; Figure S3, Supporting Information). These consistent numbers indicate minimal proliferation of grafted cells, supporting the notion that GABAergic progenitor cell transplantation offers a safer profile compared to stem cells. To validate this point, we examined Ki67 (proliferation marker) and TUNEL (apoptosis marker) staining in MGE‐grafted hippocampi at 7 and 30 DAT. Our results demonstrated low and stable rates of proliferation and apoptosis in grafted cells at both time points (Figure S4, Supporting Information). Spatially, MGE cells had migrated up to 1500 µm at 60 DAT in the CA1 area. Approximately 45% of MGE cells were situated close to the injection site, while merely ≈0.63% of MGE cells underwent a migration distance of ≈1500 µm (Figure 1E).

### 2.2 Transplanted MGE Cells Can Differentiate into Mature INs

At 60 DAT, the majority of GFP^+^ progenitors exhibited clear morphological features of mature neurons. These cells displayed extensive processes, with their cell bodies localized in the polymorphic and molecular layers, and expressed the neuronal marker NeuN (94.5% ± 3.1%) (Figure 1F; Figures S5–S7, Supporting Information). Furthermore, we found that transplanted MGE progenitors differentiated into major subtypes of INs, including parvalbumin‐positive (PV^+^) (41.3% ± 2.8%), somatostatin‐positive (SST^+^) (21.6% ± 1.2%), and neuronal nitric oxide synthase‐positive (nNOS^+^) (7.7% ± 0.8%) cells, as shown by co‐labeling with PV, SST and nNOS markers respectively (Figure 1G; Figures S8–S10, Supporting Information). Overall, these data indicate that MGE cells migrate efficiently and differentiate well within the hippocampus of adult Fmr1‐KO mice.

**Figure 2:**
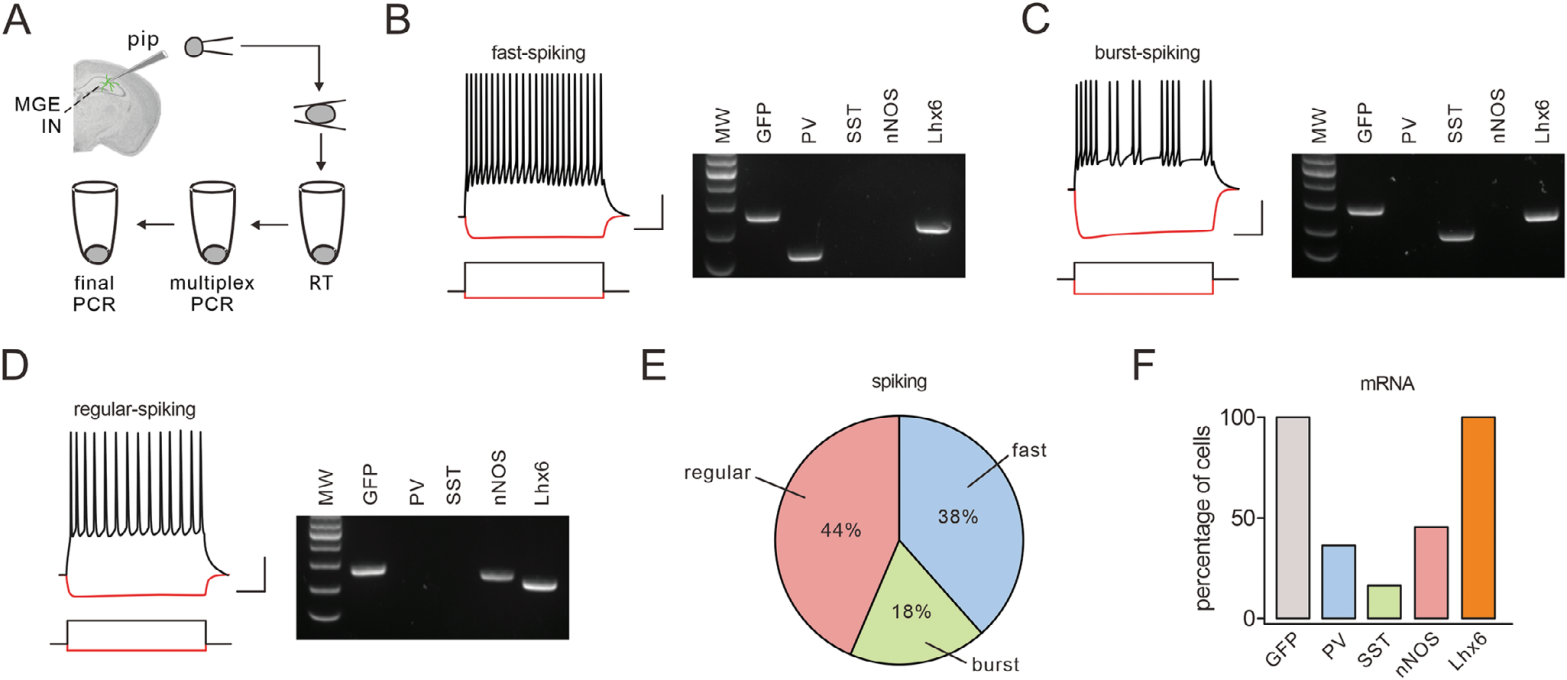
MGE cells differentiate into functional INs. A) Schematic showing scRT‐PCR in grafted MGE cells. B‐D) Example electrophysiological responses and RNA expressions of 3 MGE cells to injected currents (black: near‐maximal firing; red: hyperpolarization) from a holding potential near −70 mV. Recordings were performed from slices at 31–35 DAT. Scale bars: 20 mV/200 ms. RNA profiles were obtained from each recorded cell using scRT‐PCR. MW, molecular weight. E) Summary plot for the occurrence of each marker in recorded MGE GFP^+^ cells. Fast spiking (PV): *n* = 12. Burst spiking (SST): *n* = 6. Regular spiking (nNOS): *n* = 14. F) Occurrence of each IN subtype recorded based on firing properties (*n* = 39 cells). GFP: *n* = 39 (100%). PV: *n* = 15 (38.5%). SST: *n* = 7 (17.95%). nNOS: *n* = 17 (43.59%). Lhx6: *n* = 39 (100%).

In addition to neuronal marker expression, mature INs are expected to exhibit electrophysiological activity. To assess this, we examined the electrophysiological and molecular characteristics of GFP^+^ cells using patch‐clamp recordings combined with *post hoc* single‐cell RT‐PCR (scRT‐PCR) analysis (**Figure** **2**A). Our results demonstrated that GFP^+^ MGE cells displayed electrophysiological properties and RNA expression profiles consistent with those of mature INs in acute hippocampal slices from recipient mice (31–35 DAT). Recordings from GFP^+^ cells (*n* = 32) revealed three main subtypes of INs: fast‐spiking (38%), regular‐spiking (44%), and burst‐spiking (18%) (Figure 2B–E). scRT‐PCR analysis further confirmed that all INs expressed Lhx6, a transcription factor essential for MGE‐derived INs, along with GFP (Figure 2B–D). Meanwhile, the subsets of GFP^+^ cells expressed mRNAs encoding PV, SST, or nNOS mRNAs (Figure 2F), correlating with their distinct electrophysiological profiles. Again, these findings indicate that engrafted MGE cells differentiate into mature INs. Indeed, previous work has demonstrated that PV^+^, SST^+^, and nNOS^+^ INs exhibit fast, burst, and regular firing patterns, respectively.^[^ ^19^, ^28^ ^]^ As a negative control, we performed patch‐clamp recordings and scRT‐PCR in host INs (*n* = 4) from Fmr1‐KO mice. These cells expressed Lhx6 but not GFP (Figure S11, Supporting Information), thereby validating our scRT‐PCR results. Notably, the low proportion of nNOS^+^ cells detected via immunohistochemistry (Figure 1G) to scRT‐PCR may be attributable to the lower efficiency of nNOS antibodies.

### 2.3 MGE Cells Form Synaptic Connections with Host PNs

**Figure 3:**
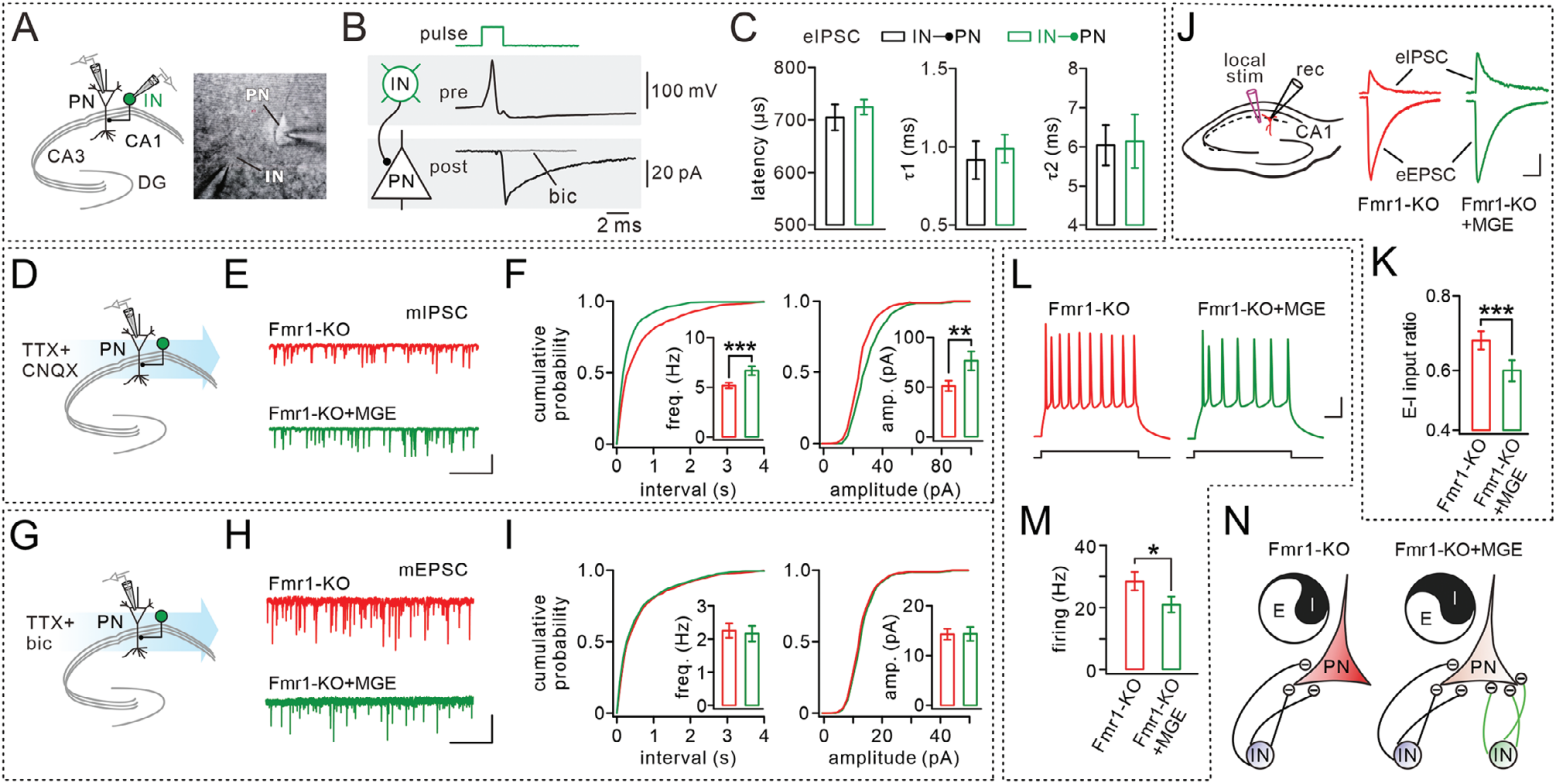
MGE cells form synaptic connections with host PNs. Note that the ages were P60 for Fmr1‐KO mice and 30 DAT for Fmr1‐KO+MGE mice. A) Schematic and image depicting dual patch‐clamp recordings from neighboring PN and IN in CA1. B) Simultaneous recording from a presynaptic IN and a postsynaptic PN. IN and PN were held in current‐ and voltage‐clamp configurations, respectively. Top: an AP in presynaptic IN. Bottom: average eEPSC from 10 individual recordings. C) Kinetics of eEPSCs of host IN‐PN (black) and MGE IN‐PN (green) synapses. D) Schematic showing mIPSC recordings from PNs. E) Example mIPSCs recorded from Fmr1‐KO and Fmr1‐KO+MGE mice. F) Cumulative plots of mIPSC amplitude and interval. G) Schematic showing mEPSC recordings from PNs. H) Example mEPSCs recorded from Fmr1‐KO and Fmr1‐KO+MGE mice. I) Cumulative plots of mEPSC amplitude and interval. J) Schematic showing eEPSC and eIPSC recorded from one PN. Note that eIPSC was increased in the Fmr1‐KO+MGE group. K) Ratios of eEPSC and eIPSC. Fmr1‐KO: 0.70 ± 0.01. Fmr1‐KO+MGE: 0.60 ± 0.02. *n* = 7 cells per group. *p* = 0.00012. L) Sample spikes from Fmr1‐KO and Fmr1‐KO+MGE neurons in response to a 300‐ms current injection (200 pA). M) Firing frequency: Fmr1‐KO, 28.5 ± 3.0 Hz; Fmr1‐KO+MGE, 21.0 ± 2.5 Hz. *n* = 6 cells per group. *p* = 0.041. N) A cartoon showing that, besides host INs (purple), INs (green) derived from MGE transplantation increased inhibitory connections to PN and reduced its excitability. See Table S2 (Supporting Information) for statistics, including *n* values, *p* values, and statistical tests. *p < 0.05. **p < 0.01. ***p < 0.001.

To investigate whether transplanted MGE cells establish synaptic connections with host CA1 PNs, we conducted double whole‐cell recordings in GFP^+^ or GFP^−^ INs and neighboring PNs in acute brain slices from 30 DAT mice (**Figure** **3**A). Our findings demonstrated that activation of MGE‐derived GFP^+^ INs could induce postsynaptic currents in adjacent PNs, which were completely abolished by bicuculline, a GABA_A_ receptor antagonist (Figure 3B). Comparisons of synaptic latency and current decay time constants revealed no significant differences between GFP^+^ and GFP^−^ INs (Figure 3C). These results indicate that MGE‐derived cells form functional inhibitory synapses with native PNs in the Fmr1‐KO hippocampus.

We further investigated the impact of MGE‐derived INs on inhibitory synaptic inputs to CA1 PNs (Figure 3D). Following transplantation, both the frequency and amplitude of miniature inhibitory postsynaptic currents (mIPSCs) in PNs were significantly increased compared to untreated Fmr1‐KO mice (Figure 3E,F), suggesting enhanced inhibitory input. We also explored the influence of transplantation on the excitatory inputs to PNs in Fmr1‐KO mice (Figure 3G). The results demonstrated that the transplantation altered neither frequency nor amplitude of miniature excitatory postsynaptic currents (mEPSCs) in PNs of Fmr1‐KO mice (Figure 3H,I), indicating that MGE cells had no effect on excitatory inputs to PNs. These results were corroborated by recording evoked EPSCs and IPSCs in the same PN from Fmr1‐KO or Fmr1‐KO+MGE mice (Figure 3J), which demonstrated that MGE transplantation led to a significant reduction in the excitatory‐inhibitory (E‐I) input ratio (Figure 3J,K), suggesting that MGE‐derived INs restore E‐I balance of Fmr1‐KO PNs. It has been reported that the intrinsic excitability of CA1 PNs is altered in FXS mice.^[^ ^29^ ^]^ Our current‐clamp recordings indicated that PNs from Fmr1‐KO+MGE mice exhibited reduced excitability compared to those from Fmr1‐KO mice (Figure 3L,M).

In summary, our findings demonstrate that MGE grafts in Fmr1‐KO mice form functional inhibitory synaptic connections with CA1 PNs and enhance inhibitory inputs without impacting excitatory inputs, thereby regulating E/I balance and intrinsic excitability of PNs (Figure 3N).

### 2.4 MGE Cell Transplantation Rescues Cognitive Deficits in Fmr1‐KO Mice

One of the hallmarks of FXS is impaired hippocampus‐dependent learning and memory.^[^ ^30^ ^]^ Therefore, we examined whether MGE cell transplantation could ameliorate cognitive deficits in Fmr1‐KO mice. For this purpose, we assigned 4 groups of mice (30 DAT) – WT, Fmr1‐KO, Fmr1‐KO+MGE, and Fmr1‐KO+dMGE (deactivated MGE) – and conducted cognitive‐related behavioral tests.

**Figure 4:**
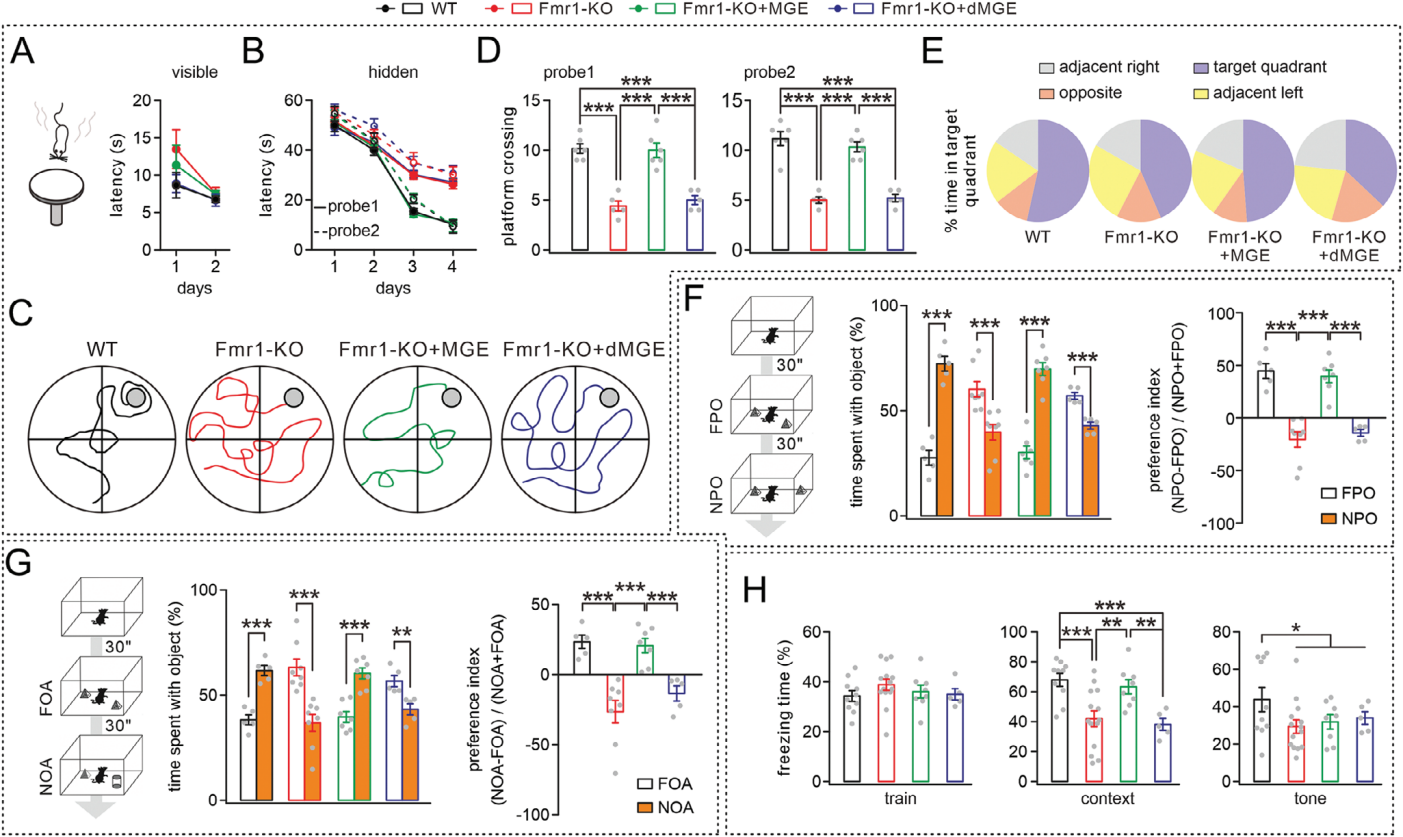
MGE transplantation rescues learning and memory in Fmr1‐KO+MGE mice. Note that the ages were P60 for WT and Fmr1‐KO mice, but 30 DAT for Fmr1‐KO+MGE and Fmr1‐KO+dMGE mice. A) In the MWM test, all mice showed a decrease in latency to escape to the visible platform. B) Fmr1‐KO+MGE mice (30 DAT) showed a marked improvement in latency to escape MWM during third and fourth days in 2 probe tests of the hidden platform. C) During the 2 probe tests, Fmr1‐KO+MGE mice made more target platform crossings. D) Example locomotion tracking plots showing total path length on day 4 of the hidden platform task. E) Fmr1‐KO+MGE mice (30 DAT) showed a preference for the target quadrant compared to Fmr1‐KO (P60) or Fmr1‐KO+dMGE (30 DAT) mice. F) Left: schematic showing object cognition test. Middle: bar graphs show percentages of time spent with FPO and NPO. Right: preferential indexes to NPO. G) Left: schematic showing location cognition test. Middle: bar graphs show percentages of time spent with FOA and NOA. Right: preferential indexes to NOA. H) Freezing time of 4 groups of mice during baseline training (left panel) and after CFC (middle panel) and TFC (right panel) tests. See Table S3 (Supporting Information) for statistics, including *n* values, *p* values, and statistical tests. *p < 0.05. **p < 0.01. ***p < 0.001.

First, the mice underwent the Morris water maze (MWM) test, which requires visual cues to locate a hidden platform. Initially, a visible platform was positioned above the water surface for the mice to locate, and their latency to the platform as well as swimming speed was recorded. We found that all groups of mice efficiently navigated toward the platform with similar swimming speeds after 2 days of training, indicating normal visual and motor functions (**Figure** **4**A; Table S3, Supporting Information). Subsequently, the platform was submerged below the water's surface, which requires rodents' use of spatial memory for its location. In separate probe tests with platforms placed in opposing quadrants over four‐day training sessions, both WT and Fmr1‐KO+MGE mice showed reduced times in locating it during probe 1 (Figure 4B). Fmr1‐KO or Fmr1‐KO+dMGE mice also displayed decreased search time; however, their performance on days three and 4 notably lagged behind WT or Fmr1‐KO+MGE mice (Figure 4B), indicating poorer learning ability. During probe 2 in an opposing quadrant, all groups demonstrated the same performances as seen in probe 1. The discernible disparities in spatial learning capabilities among these groups were depicted by their swimming paths on the final day of training (Figure 4C). After removing the platform, both the number of times mice crossed over the platform location and the time spent in each quadrant were recorded. In both probes 1 and 2, Fmr1‐KO and Fmr1‐KO+dMGE mice showed significantly fewer crossings compared to WT and Fmr1‐KO+MGE mice (Figure 4D). Further analyses revealed that Fmr1‐KO mice and Fmr1‐KO+dMGE mice spent notably less time in the quadrant with the platform than WT and Fmr1‐KO+MGE mice (Figure 4E). Taken together, these findings suggest an improvement in spatial learning and memory among Fmr1‐KO mice following the MGE graft.

Second, 4 groups of mice were subjected to an object location test (OLT), which relies on hippocampal function to assess cognitive aptitude in the immediate environment.^[^ ^31^ ^]^ Animals were examined regarding their proficiency in identifying an object displaced to a new location (Figure 4F). Our findings disclosed that WT and Fmr1‐KO+MGE mice sensed a minor change in the environment by expending a considerable amount of exploration when the object was displaced to a new location (novel place object, NPO) compared to when the object was in the familiar place (familiar place object, FPO) (Figure 4F). Differently, Fmr1‐KO and Fmr1‐KO+dMGE mice demonstrated impairment in this task, displaying similar levels of interest toward both NPO and FPO (Figure 4F). We also investigated the impact of MGE graft on recognition learning by employing a novel object recognition (NOR) test (Figure 4G), which partially relies on hippocampal function.^[^ ^31^ ^]^ Our findings suggest that both WT and Fmr1‐KO+MGE mice exhibited normal recognition, as evidenced by their increased exploration time in the novel object area (NOA) compared to the familiar object area (FOA) (Figure 4G). In contrast, Fmr1‐KO and Fmr1‐KO+dMGE mice exhibited comparable impairment in recognition memory by exploring NOA and FOA nearly equally (Figure 4G).

Third, contextual fear conditioning (CFC) was employed to assess memory retention, which is significantly impaired in Fmr1‐KO mice.^[^ ^32^ ^]^ During fear conditioning, all 4 groups of mice displayed comparable levels of freezing (Figure 4H), indicating intact sensory perception. Subsequent testing conducted 24 h after fear conditioning revealed reduced freezing in Fmr1‐KO and Fmr1‐KO+dMGE mice, while WT and Fmr1‐KO+MGE mice exhibited significantly increased freezing (Figure 4H). These results suggest that the MGE graft effectively reverses impaired CFC learning in Fmr1‐KO mice. In addition, we performed a hippocampal‐independent tone fear conditioning (TFC) test.^[^ ^33^ ^]^ Our findings indicated that compared to WT mice, Fmr1‐KO, Fmr1‐KO+MGE, and Fmr1‐KO+dMGE mice exhibited decreased freezing, further suggesting that MGE graft primarily impacts hippocampal function.

Given that both FXS patients and mouse models exhibit autistic behaviors,^[^ ^34^ ^]^ we next assessed social learning across 4 groups of mice using a three‐chamber interaction test. In the initial phase, all mice groups consistently preferred S1 mouse over the empty cage, indicating intact sociability (Figure S12A, Supporting Information). Subsequently, during the social novelty phase, which assesses social cognition, we noted a significantly higher preference index for S2 mouse over S1 mouse in WT and Fmr1‐KO+MGE mice, but not in Fmr1‐KO or Fmr1‐KO+dMGE mice (Figure S12B, Supporting Information). Furthermore, WT and Fmr1‐KO+MGE mice displayed prolonged sniffing times toward S2 mice compared to Fmr1‐KO or Fmr1‐KO+dMGE mice (Figure S12B, Supporting Information). These results suggest improved social memory following MEG transplantation in Fmr1‐KO mice.

Finally, we assessed the impact of MGE transplantation on the emotional behaviors of Fmr1‐KO mice. The open field test revealed that WT and Fmr1‐KO+MGE mice displayed increased activity in the inner zone, while Fmr1‐KO and Fmr1‐KO+dMGE mice exhibited a preference for the outer zone (Figure S13A, Supporting Information). Furthermore, no significant difference in overall locomotion was observed across the 4 groups. These findings suggest that MGE transplantation alleviates anxiety in Fmr1‐KO mice. Repetitive behavior, a hallmark feature of Fmr1‐KO mice, was assessed through grooming, marble burying, and T‐maze tasks. Fmr1‐KO and Fmr1‐KO+dMGE mice exhibited significantly more marble burying than WT and Fmr1‐KO+MGE mice (Figure S13B, Supporting Information). However, there were no significant differences in repetition scores among the 4 groups of mice in the T‐maze test (Figure S13E,F, Supporting Information). Moreover, there were no variations in interrupted bouts or grooming time during the water spray‐induced self‐grooming test (Figure S13G,H, Supporting Information). These findings suggest that the impact of MGE transplantation on repetitive behaviors in Fmr1‐KO mice is inconsistent, possibly due to varying neural mechanisms underlying different stereotypic behaviors. We also investigated the effect of the MGE graft in the hippocampus on motor activity. Fmr1‐KO mice showed limited motor learning over 8 sessions in the rotarod test: their latency to fall was shorter than WT mice at late stages. MGE transplantation did not improve the motor deficits of Fmr1‐KO mice (Figure S13I, Supporting Information).

### 2.5 MGE Transplantation Restores Impaired LTP and GluA1 Trafficking in Fmr1‐KO Mice

**Figure 5:**
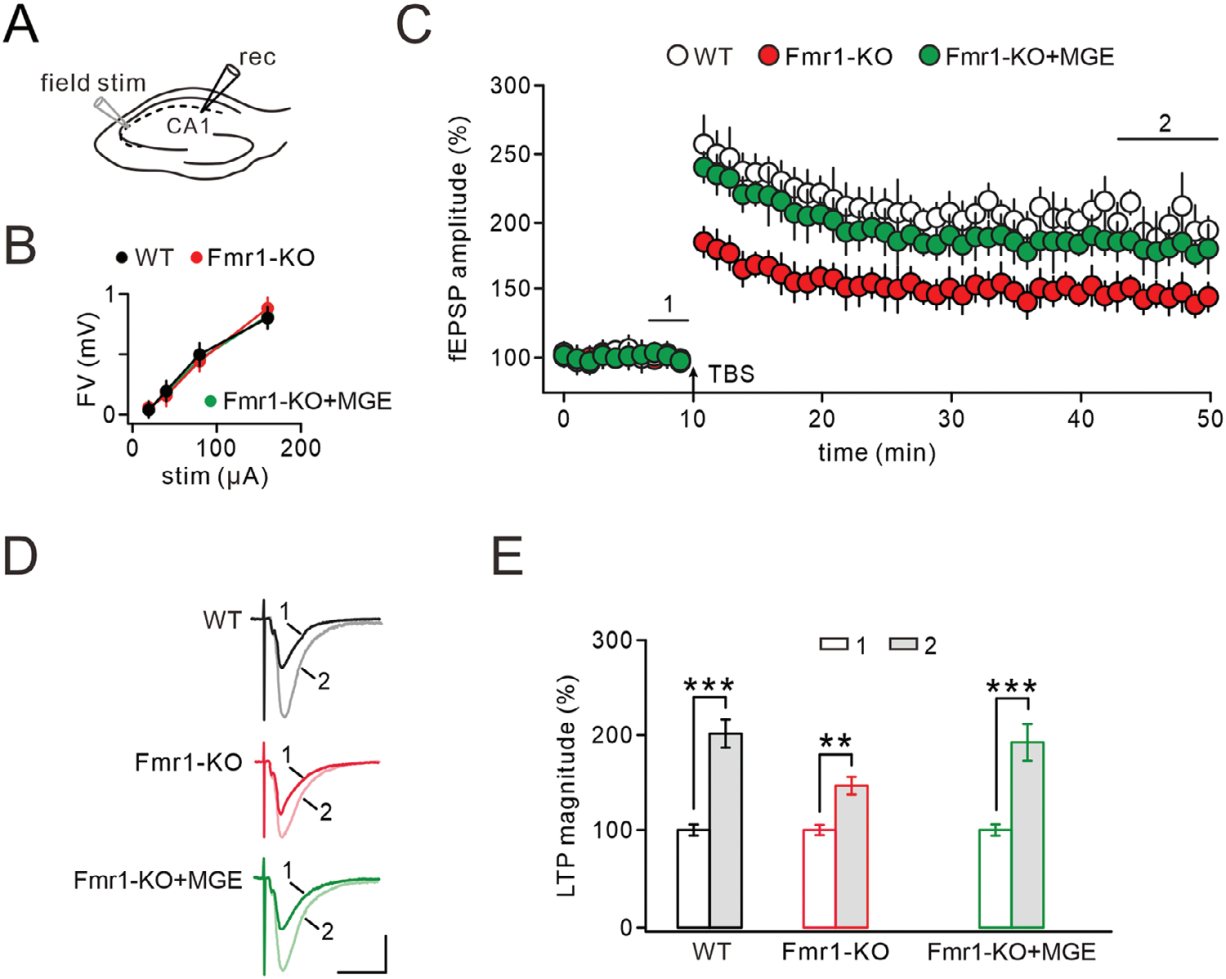
MGE transplantation restored LTP expression in Fmr1‐KO mice. Note that the ages were P60 for WT and Fmr1‐KO mice, but 30 DAT for Fmr1‐KO+MGE mice. A) Schematic diagram illustrating the stimulation of the SC using a concentric bipolar electrode and the recording of fEPSP in the CA1. B) Fiber volley (FV) induced by different stimulation intensities in 3 groups of mice. C) Time course of LTP before and after TBS stimulation in 3 groups of mice. D) Representative fEPSC traces from 3 groups of animals, with sampling time points 1 and 2 as indicated in C. E) Mean values of LTP amplitude in WT, Fmr1‐KO, and Fmr1‐KO+MGE groups. See Table S6 (Supporting Information) for statistics, including *n* values, *p* values, and statistical tests. **p < 0.01. ***p < 0.001.

To investigate the mechanism underlying the enhancement of cognition in Fmr1‐KO mice following the MGE graft, we conducted field EPSP (fEPSP) recording to assess LTP expression in the hippocampus of WT, Fmr1‐KO, and Fmr1‐KO+MGE mice. Stimulation was applied to the Schaffer collateral (SC) pathway using a concentric bipolar electrode while fEPSPs were recorded in the CA1 area (**Figure** **5**A). Our findings revealed consistent fiber volley across all three mouse groups in response to varying stimulation intensities (Figure 5B). Specifically, theta‐burst stimulation (TBS) induced robust LTP of fEPSP in WT mice but showed a significant reduction in Fmr1‐KO mice; however, this impairment was largely restored by MGE graft (Figure 5C). The maintained LTP magnitudes were 199.9% ± 14.2% for WT mice, 145.5% ± 9.0% for Fmr1‐KO mice, and 189.2% ± 21.8% for Fmr1‐KO+MGE mice (Figure 5D,E).

**Figure 6:**
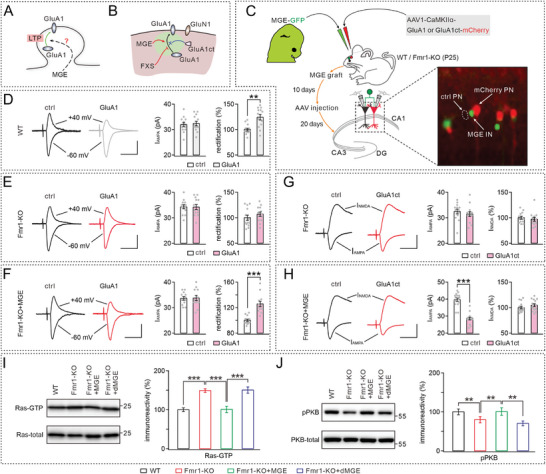
MGE cells stimulate synaptic GluA1 trafficking in Fmr1‐KO mice. Note that the ages were P55 for WT and Fmr1‐KO mice, but 30 DAT for Fmr1‐KO+MGE and Fmr1‐KO+dMGE mice. A) Schematic showing a possible role of MGE cells on LTP. B) Schematic drawing outlines in vivo experimental design using GluA1ct. C) MGE‐derived GABAergic progenitor cells were transplanted into WT or Fmr1‐KO hippocampus and differentiated to INs (green fluorescence). GluA1 or GluAct was then expressed specifically to PNs using CaMKIIα promoter‐based AAV‐mCherry virus (red fluorescence). Whole‐cell recordings were made in neighboring infected and non‐infected PNs. D) Evoked AMPA responses recorded from mCherry‐ (ctrl) or GluA1‐mCherry‐expressing WT PNs. Bar graphs show I_AMPA_ amplitudes and rectification of I_AMPA_ normalized to the control. I_AMPA_: 31.9 ± 0.9 pA (ctrl; *n* = 13) and 32.4 ± 0.9 pA (GluA1; *n* = 13; *p* = 0.71). Rectification: 100% ± 2.8% (ctrl; *n* = 13) and 124.2% ± 5.6% (GluA1; *n* = 13; *p* = 0.00048). E) Evoked AMPA responses of control or GluA1‐expressing Fmr1‐KO PNs. I_AMPA_: 34.3 ± 0.8 pA (ctrl; *n* = 13) and 34.2 ± 1.0 pA (GluA1; *n* = 13; *p* = 0.95). Rectification: 100% ± 2.1% (ctrl; *n* = 13) and 102.8% ± 1.8% (GluA1; *n* = 13; *p* = 0.31). F) Evoked AMPA responses of control or GluA1‐expressing Fmr1‐KO+MGE PNs. I_AMPA_: 32.6 ± 0.8 pA (ctrl; *n* = 13) and 32.8 ± 1.1 pA (GluA1; *n* = 13; *p* = 0.90). Rectification: 100% ± 2.1% (ctrl; *n* = 13) and 125.8% ± 4.5% (GluA1; *n* = 13; *p* < 0.001). G) I_AMPA_ and I_NMDA_ recorded from control or GluA1ct‐expressing PNs from Fmr1‐KO mice. Bar graphs show average amplitudes of I_AMPA_ and percentage changes of I_NMDA_ to the control. I_AMPA_: 32.5 ± 1.0 pA (ctrl; *n* = 13) and 31.5 ± 1.0 pA (GluA1; *n* = 13; *p* = 0.46). Change of I_NMDA_: 100% ± 1.2% (ctrl; *n* = 13) and 98.8% ± 1.6% (GluA1; *n* = 13; *p* = 0.56). H) I_AMPA_ and I_NMDA_ recorded from control or GluA1ct‐expressing Fmr1‐KO+MGE PNs. I_AMPA_: 39.7 ± 1.0 pA (ctrl; *n* = 13) and 28.5 ± 0.9 pA (GluA1; *n* = 13; *p* < 0.001). Change of I_NMDA_: 100% ± 2.6% (ctrl; *n* = 13) and 104.6% ± 2.9% (GluA1; *n* = 13; *p* = 0.23). I) Blots of GTP‐bound Ras and total Ras in CA1. Bar graphs show relative amounts of Ras‐GTP. J) Blots of phos‐PKB and total PKB in CA1. Bar graphs show relative amounts of phos‐PKB. See Table S7 (Supporting Information) for more statistics, including *n* values, *p* values, and statistical tests. **p < 0.01. ***p < 0.001.

Next, we investigated how the engrafted MGE cells altered LTP induction in Fmr1‐KO mice. Previous studies have established the crucial role of GluA1‐containing AMPA receptors (AMPARs) trafficking in hippocampal LTP.^[^ ^35^ ^]^ Therefore, we hypothesized that MGE cells may enhance GluA1 trafficking in Fmr1‐KO mice (**Figure** **6**A). To determine whether synaptic delivery of endogenous GluA1 is impaired in Fmr1‐KO mice and restored by MGE graft, we evaluated the effects of the cytoplasmic termini of GluA1, known as GluA1ct (Figure 6B), which serves as a dominant negative construct and specifically inhibits the synaptic trafficking of GluA1‐containing AMPARs.^[^ ^36^, ^37^ ^]^

GluA1‐mCherry or GluA1ct‐mCherry, carried by AAV1‐CaMKIIα virus that preferentially infects excitatory neurons,^[^ ^38^ ^]^ was expressed in the hippocampus of recipient WT or 30 DAT Fmr1‐KO+MGE mice (Figure 6C). Twenty days after the injection, we recorded the evoked AMPAR and NMDAR‐mediated currents in adjacent expressing or non‐expressing PNs in acute hippocampal slices (Figure 6C). In whole‐cell patch clamp recordings from WT slices, PNs expressing GluA1 showed a ≈30% increase in the rectification of AMPA responses compared to neighboring non‐expressing neurons (Figure 6D), suggesting an enhanced synaptic delivery of rectifying GluA1 subunits.^[^ ^39^, ^40^ ^]^ Conversely, both GluA1‐expressing and non‐expressing PNs displayed similar amplitudes of AMPA responses (Figure 6D), indicating normal delivery of endogenous GluA1 in the nonexpressing PNs.

For comparison, GluA1 was expressed in CA1 PNs of both Fmr1‐KO and Fmr1‐KO+MGE mice, and AMPA currents and rectification were examined. Similarly, the amplitude of AMPAR responses in GluA1‐expressing and non‐expressing PNs was found to be the same in both Fmr1‐KO (Figure 6E) and Fmr1‐KO+MGE mice (Figure 6F). However, GluA1‐expressing PNs from Fmr1‐KO+MGE mice showed an enhanced (≈80%) rectification of AMPAR responses (Figure 6F), a phenomenon not observed in GluA1‐expressing PNs from Fmr1‐KO mice (Figure 6E). The unchanged AMPA responses in Fmr1‐KO mice were consistent with previous findings.^[^ ^39^, ^41^ ^]^ These results suggest that the absence of Fmr1 impedes the synaptic transportation of GluA1, a deficit that can be restored through MGE graft.

To further clarify the impact of MGE transplantation on GluA1 trafficking, we introduced GluA1ct into CA1 PNs of both Fmr1‐KO and Fmr1‐KO+MGE mice. In comparison to neighboring neurons lacking GluA1ct expression in Fmr1‐KO slices, those expressing GluA1ct exhibited similar AMPA and NMDA currents (Figure 6G), indicating that synaptic transport of GluA1 in these neurons was influenced by Fmr1 loss and thus not susceptible to inhibition by GluA1ct. However, in Fmr1‐KO+MGE mice, AMPA currents in GluA1ct‐expressing PNs were significantly reduced by ≈50%, while NMDA currents remained unchanged (Figure 6H). This finding suggests that MGE graft restores synaptic transport of GluA1, which had been blocked by expressing GluA1ct.

### 2.6 MGE Transplantation Stimulates Ras‐PKB Signaling in Fmr1‐KO Mice

To investigate the mechanism underlying the action of MGE cells on synaptic GluA1 transport, we analyzed the Ras‐PKB signaling pathway, which plays a crucial role in regulating synaptic GluA1 trafficking.^[^ ^37^, ^39^, ^40^, ^42^ ^]^ We assessed the levels of active Ras‐GTP (GTP‐bound Ras) and phosphorylated PKB (p‐PKB), both of which are dependent on mouse activity state, in the CA1 region isolated from WT, Fmr1‐KO, Fmr1‐KO+MGE, and Fmr1‐KO+dMGE mice at 7:00 pm. The results demonstrated a significantly elevated level of Ras‐GTP in Fmr1‐KO and Fmr1‐KO+dMGE mice compared to WT or Fmr1‐KO+MGE mice (Figure 6I), indicating decreased Ras‐GTP in Fmr1‐KO mice that could be rescued by MGE transplantation. Further analysis revealed a markedly reduced level of p‐PKB‐S304 in Fmr1‐KO mice compared to WT mice (Figure 6J), indicative of impaired p‐PKB in the absence of Fmr1. MGE transplantation but not dMGE reversed this reduction in p‐PKB observed in Fmr1‐KO mice (Figure 6J). These findings indicate that MGE transplantation can restore impaired Ras‐PKB signaling pathways in Fmr1‐KO mice, thereby providing a mechanistic basis for its effect on synaptic GluA1 transport.

### 2.7 MGE Transplantation Rescues Abnormal Neural Oscillations in Fmr1‐KO Mice

The temporal coordination of neuronal activity is manifested in the rhythmic oscillations of the local field potential (LFP).^[^ ^43^ ^]^ Neural oscillations have been linked to cognitive and mechanistic processes throughout the brain, encompassing neuronal communication and precise spike timing of neuronal groups.^[^ ^44^, ^45^, ^46^ ^]^ Abnormal spike‐phase locking and phase‐amplitude coupling (PAC) have been detected in FXS mice,^[^ ^47^ ^]^ which is associated with impaired E‐I balance.^[^ ^8^, ^48^ ^]^ As a result, we examined the temporal coordination of hippocampal oscillatory rhythms in WT, Fmr1‐KO, and Fmr1‐KO+MGE mice.

**Figure 7:**
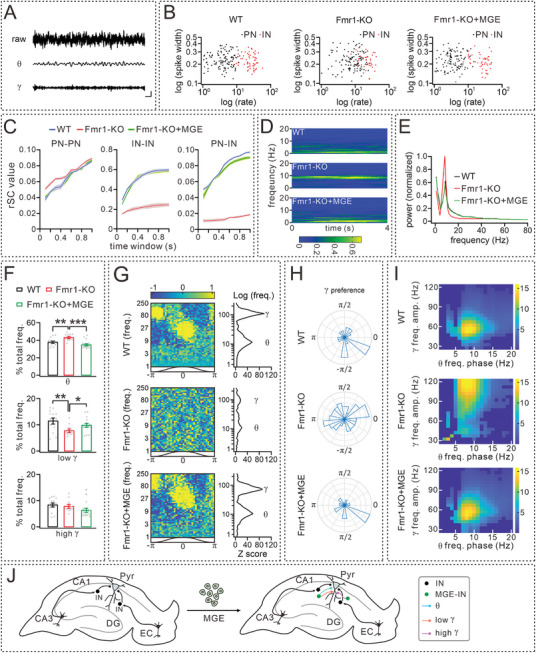
MGE cell transplantation restores neural oscillations in Fmr1‐KO mice. Note that the ages were P60 for WT and Fmr1‐KO mice, but 30 DAT for Fmr1‐KO+MGE mice. A) Example LFP raw, θ (4–12 Hz) and γ (20–100 Hz) bands. Scale bars: 4 s / 50 µV. B) Spike half‐width and mean firing rate of PNs and INs. PN firing: 4.73 ± 0.15 Hz (WT); 9.72 ± 0.22 Hz (Fmr1‐KO); 4.26 ± 0.15 Hz (Fmr1‐KO+MGE); IN firing: 26.13 ± 0.39 Hz (WT); 23.27 ± 0.52 Hz (Fmr1‐KO); 28.17 ± 0.48 Hz (Fmr1‐KO+MGE). C) Spike count correlations between PNs, INs, and mixed PN and IN pairs across varying time windows. Gray bar marks significant differences between genotypes. D) Time frequency power spectra of LFP recorded in CA1, during the first 4 s of each continuous recorded segment in which animals moved above threshold speed (3 cm ^−1 ^s). E) Full spectrum CA1 LFP power. Gray bar marks significant differences between genotypes. F) Relative LFP powers in θ band (4–12 Hz), low γ band (20–50 Hz) and high γ band (55–100 Hz). G) Spike phase modulation of PNs as a function of instantaneous LFP frequency in 3 groups. Left: Color bar shows normalized Z‐scored firing rate. Right: median Rayleigh's Z statistic for each population as a function of LFP frequency. H) Example distributions of γ phase preference of PNs in 3 groups. I) Standard PAC comodulogram depicting the amplitudes of oscillations at frequencies ranging from 25 to 125 Hz being modulated by the phase of oscillations at frequencies ranging from 1 to 20 Hz. J) Schematic illustration depicting the proposed mechanisms through which differentiated MGE INs integrate into the hippocampal neural circuit (SC and TA pathways) and modulate θ and γ oscillations. See Tables S8–S10 (Supporting Information) for statistics, including *n* values, *p* values, and statistical tests. **p < 0.01. ***p < 0.001.

Mice were habituated to an open arena, and tetrode recordings were performed in CA1 region while they freely explored the arena. The epochs during which the mice exhibited immobility were compiled and analyzed. Spike‐sorting analysis of the recorded LFP demonstrated that all mice exhibited strong θ band (4–12 Hz) activity with nested γ band (20–100 Hz) (**Figure** **7**A; Figure S14A, Supporting Information), the typical features of hippocampal LFP.^[^ ^49^ ^]^ We identified 310 putative PNs (WT 114, Fmr1‐KO 115, and Fmr1‐KO+MGE 112) and 146 putative INs (WT 61, Fmr1‐KO 34, and Fmr1‐KO+MGE 51). PNs and INs exhibited distinct firing characteristics, including waveform, firing interval, and autocorrelation, enabling easy differentiation (Figure S14B,C, Supporting Information; also see Method for autocorrelation analysis). No difference was found among 3 groups of mice in the spike waveforms of either PNs or INs. However, firing rates showed genotype‐specific differences, as Fmr1‐KO mice exhibited increased PN firing and decreased IN firing compared to WT mice (Figure 7B). Importantly, MGE transplantation effectively reversed the alteration by enhancing IN firing and reducing PN firing in Fmr1‐KO+MGE mice (Figure 7B).

Next, we evaluated whether Fmr1 deficiency triggers hippocampal network aberration using spike‐count correlation (rSC), a measure of common variance between 2 neurons.^[^ ^50^, ^51^ ^]^ Since rSC can be affected by time window width,^[^ ^52^, ^53^ ^]^ we studied rSCs across a wide range of time windows (from 0.1 to 1 s) with a bin of 0.1 s. Within this window, there was a monotonic and rapid increase in rSC values of PN‐PN, IN‐IN, and IN‐PN in WT mice (Figure 7C). Differently in Fmr1‐KO mice, rSCs exhibited a rapid increase in PN‐PN, while showing a significantly slow increase in both IN‐IN and PN‐IN across the range of counting windows (Figure 7C). Notably, MGE transplantation markedly restored IN‐IN and PN‐IN rSCs in Fmr1‐KO mice, nearly approaching the level of WT mice (Figure 7C).

We then examined the impact of MGE transplantation on firing frequency bands in the Fmr1‐KO hippocampus. Analyzing 0–20 Hz bands over a 4‐s period revealed a significant increase and more concentrated θ wave firings in Fmr1‐KO mice compared to WT mice (Figure 7D). Moreover, full‐spectrum analysis showed a significant reduction in low γ bands (20‐50 Hz), with high γ bands (55–100 Hz) unaltered (Figure 7E). These findings were consistent across animals, as reflected in group averages (Figure 7F). Notably, both individual animal analysis and averaged measurements confirmed that MGE transplantation effectively restored distorted θ and low γ oscillations in Fmr1‐KO mice (Figure 7D–F). It is known that θ and low γ bands are present in the communication between CA1 and CA3, meanwhile, high γ bands are present between CA1 and EC.^[^ ^54^, ^55^, ^56^ ^]^ Therefore, these results support that MGE transplantation rescues the defects in the SC pathway, which is essential for hippocampal function.

We next examined spike‐phase locking of PNs to neural oscillations, which displays significant θ and γ phase preference.^[^ ^55^, ^57^ ^]^ In WT mice, a larger proportion of PNs were recruited to lock in γ oscillation (Figure 7G,H), consistent with previous reports.^[^ ^58^, ^59^ ^]^ In contrast, PNs from Fmr1‐KO mice exhibited a significant loss of locking probability (Figure 7G,H), suggesting that the deficiency of FMRP disrupts the functional encoding of PNs through dysregulated firing patterns and disconnects hippocampal oscillation and neuronal activity. Notably, MGE transplantation could markedly restore hippocampal rhythms of Fmr1‐KO mice, with metrics showing enhanced powers of θ and γ bands simultaneously (Figure 7G,H). These results support that MGE transplantation rescues the defects in the TA pathway.

It has been suggested that there is a relationship between γ oscillation amplitude and θ oscillation phase, and such cross‐frequency coupling significantly increases during cognitive learning.^[^ ^60^, ^61^ ^]^ To investigate this, we employed a standard θ‐γ PAC algorithm in epochs with matched and filtered LFP envelopes in order to detect coupling strength.^[^ ^58^ ^]^ Initially, LFP recordings from the hippocampus were processed using MatLab to extract the amplitudes and phases of θ and γ oscillations (Figure S15A, Supporting Information). Subsequently, the correlation between θ oscillation (1–20 Hz) phase and γ oscillation (25–120 Hz) amplitude was computed. According to previous work,^[^ ^61^ ^]^ we applied the Hilbert Transform Y_θ_ = H(X_θ_) converts real‐valued signal into analytic signal of the form *Z*_θ_(*t*) = X_θ_(*t*) + iY_θ_(*t*) = *A*_θ_(*t*)e^iΨ^_θ_^(^ *^t^* ^)^, where *A*_θ_(*t*) is θ amplitude envelope and Ψ_θ_(t) is θ analytic phase time series (Figure S15B, Supporting Information). Similarly, we constructed the analytic signal for γ band: *Z*_γ_(*t*) = X_γ_(*t*) + iY_γ_(*t*) = *A*_γ_(*t*)e^iΨ^_γ_^(^ *^t^* ^)^ (Figure S15C, Supporting Information). To determine the modulation index (*MI*), we constructed a composite complex‐valued signal by combining the amplitude time series of one frequency with the phase time series of the other. The composite signals combined γ amplitude with θ phase: *Z*(*t*) = A_γ_(t)e^iΨ^_θ_(*t*).^[^ ^61^ ^]^ For each sample point, this composite signal took on some particular value in the complex plane (Figure S15D,E, Supporting Information), and *Z*(*t*) and *MI*_raw_ were calculated according to Equations (6) and (7) (See Experimental Section).^[^ ^61^ ^]^ Next, a Gaussian fit was applied to 200*MI*_raw_ to derive the mean value μ and variance value σ of the Gaussian distribution, and *MI* is computed using Equation (8) (See Experimental Section; Figure S15F, Supporting Information). It was noted that a strong coupling occurred in θ band of WT and Fmr1‐KO+MGE mice, but was much reduced in Fmr1‐KO mice, particularly at the frequency between 1 to 20 Hz (Figure S15F, Supporting Information).

Further PAC comodulogram analysis indicated that hippocampal LFPs from WT mice exhibited distinct modulation of both low‐ and high‐γ amplitudes by θ phases (Figure 7I). In contrast, such coupling between low‐ or high‐γ amplitudes and θ rhythms remarkably reduced in Fmr1‐KO mice (Figure 7I). These results reveal that Fmr1‐KO mice exhibit a general dysfunction of temporal integration in local circuits. Interestingly, with grafted MGE cells, the emergence of γ bursts was more decentralized within a single θ cycle in Fmr1‐KO mice (Figure 7I). Overall, our data indicate that MGE graft re‐coordinates dysfunctional γ oscillations and local PAC in Fmr1‐KO mice, which further acts on hippocampus‐related memory processing.

## 3 Discussion

In this study, we demonstrate that GABAergic progenitor cells isolated from embryonic mouse MGE can be successfully transplanted into the hippocampus of Fmr1‐KO mice, achieving a survival rate of ≈30%. The transplanted cells exhibited robust migratory capabilities, differentiated into diverse IN subtypes, and formed functional synaptic connections with host PNs. These connections effectively modulated synaptic inputs and restored the E‐I balance of PNs. Additionally, MGE transplantation improved the activity of the Ras‐PI3K signaling pathway in PNs, thereby rescuing impaired synaptic transport of GluA1 and restoring synaptic plasticity (Figure S16, Supporting Information). Our in vivo electrophysiological recordings revealed that MGE transplantation re‐coordinated hippocampal neural oscillations in Fmr1‐KO mice. This was evidenced by increased spike‐count correlation between IN and PN as well as the reversal of abnormal θ and γ oscillations caused by the absence of Fmr1. Furthermore, MGE transplantation improved the phase locking and PAC of θ and γ oscillations among CA1 PNs, elucidating a circuit‐level mechanism through which the hippocampal function was restored in Fmr1‐KO mice (Figure S16, Supporting Information). Behavioral analyses further supported that MGE cells transplanted into the hippocampus significantly ameliorated deficits in spatial memory, fear memory, object recognition, and emotional memory in Fmr1‐KO mice, without affecting repetitive behaviors and motor activity. In conclusion, our findings underscore promising clinical implications of transplanting MGE‐derived GABAergic progenitor cells for the treatment of FXS and also open a new path for treating other psychiatric disorders.

### 3.1 Treatment of FXS by GABAergic Progenitor Cell Transplantation

We demonstrate that GABAergic progenitor cells derived from the MGE differentiate into PV^+^, SST^+^, and nNOS^+^ INs in the hippocampus of Fmr1‐KO mice. The hippocampus hosts a diverse population of endogenous MGE‐derived INs, including PV^+^ basket cells, PV^+^ chandelier cells, SST^+^ molecular layer INs, nNOS^+^ glial cells, and nNOS^+^ Ivy cells.^[^ ^62^ ^]^ Additionally, cholecystokinin‐positive INs originating from the CGE and calretinin‐positive INs are also present in the hippocampus.^[^ ^63^, ^64^ ^]^ Thus, it is plausible that GABAergic progenitor cells from CGE might differentiate into other IN subtypes upon transplantation. Further investigation should explore the potential effects of CGE‐derived IN subtypes and assess the therapeutic outcomes of co‐transplanting MGE‐ and CGE‐derived cells on FXS.

Mutations in Fmr1 lead to disrupted expression of neuronal receptors and synaptic proteins, resulting in impaired synaptic transmission and plasticity.^[^ ^4^, ^5^, ^7^, ^11^, ^12^ ^]^ Despite extensive efforts to develop drug therapies targeting synaptic receptors such as mGluR1/5 agonists/inhibitors or GABAR modulators, clinical outcomes have been largely unsuccessful.^[^ ^13^ ^]^ Although multiple factors may contribute to these failures, the lack of comprehensive regulatory effects in drug treatments likely plays a critical role. In contrast, progenitor cell transplantation offers several advantages for enhancing brain functions. These include the generation of diverse IN types, the formation of new neural circuits in coordination with endogenous neurons, and the provision of intricate, multi‐level cellular and molecular regulatory mechanisms. Collectively, these features enable progenitor cell transplantation to significantly mitigate the effects of Fmr1 mutations and address the complex neurobiological deficits of FXS.

Transcription factors are key regulators of IN differentiation and development. GABAergic progenitor cells derived from the MGE initially express NKX2.1, which is replaced by Lhx6 upon exiting the cell cycle. The expression of NKX2.1 declines while Lhx6 level remains stable post migration.^[^ ^25^ ^]^ These distinct transcriptional dynamics during development provide a precise modulatory framework for directing IN subtype differentiation. However, this level of transcriptional control was not addressed in the present study. Future studies should focus on optimizing the expression of transcription factors during transplantation to achieve a controlled distribution of IN subtypes, thereby refining the therapeutic potential of progenitor cell‐based interventions in FXS.

### 3.2 Spatial Distribution and Functional Correlation of MGE Graft‐Derived INs

Our findings demonstrate that MGE‐derived cells transplanted into the hippocampus exhibit robust migration across the hippocampal subregions and differentiate in INs with characteristic axonal and dendritic morphologies. The mechanism underlying this efficient migration remains unclear but may involve neural factors secreted by PNs.

Hippocampal INs are distinguished by diverse morphological and molecular characteristics. For instance, PV^+^ INs can differentiate into basket cells, chandelier cells, and bilayer cells. Each IN has a unique degree of innervations to PNs, influencing the dendrites, axons, or somata of PNs and exerting differential effects on synaptic plasticity.^[^ ^65^ ^]^ Our patch‐clamp recordings confirm that differentiated INs establish functional inhibitory synapses with PNs. These newly formed synapses exhibit properties comparable to native inhibitory synapses, effectively modulating PN excitability and synaptic plasticity. While these results illustrate the functional integration of MGE‐derived INs, a direct correlation between the spatial location, morphology, and functional roles of differentiated INs remains inconclusive. Most of the INs analyzed in our experiments were located within the central region of CA1, limiting our ability to explore spatially dependent functional heterogeneity in detail.

In addition, MGE transplantation significantly improved θ and γ oscillations disrupted by Fmr1 loss. Previous studies suggest that θ and low γ oscillations are modulated by INs within the SC pathway, while high γ waves are regulated by INs in the TA pathway originating from LEC.^[^ ^66^ ^]^ Given the widespread distribution of differentiated INs throughout CA1, it is plausible that transplanted MGE cells modulate PNs through both SC and TA pathways. This functional modulation may involve distinct subtypes of INs at specific locations (Figure 7J). Future studies should employ high‐resolution imaging to further characterize the morphology and spatial distribution of transplanted INs and elucidate their precise connectivity with PNs.

### 3.3 MGE Transplantation Modulates GluA1 Trafficking via Ras‐PI3K Signalling

While the scientific and medical communities continue to uncover the complexities of stem cell biology, this study provides evidence supporting the rational and evidence‐based application of neural progenitor cells. Specifically, we demonstrate that MGE transplantation restores normal Ras‐PKB signaling in Fmr1‐KO mice. As FMRP regulates the synthesis of Ras‐PI3K/PKB signaling molecules, its absence inevitably leads to defects in synaptic Ras transport and/or function.^[^ ^39^, ^67^ ^]^ Dysregulated Ras‐PI3K/PKB signaling is implicated in several characteristics of FXS, including spinal abnormalities, facial deformities, and the paradoxical reduction in cancer incidence observed in these patients.^[^ ^68^, ^69^, ^70^ ^]^

The Ras‐PI3K‐PKB signaling pathway is integral to synaptic GluA1 trafficking and hippocampal LTP.^[^ ^71^ ^]^ Previous studies have shown that FDA‐approved psychoactive drugs targeting serotonin or dopamine can modulate this pathway and influence hippocampal neuronal activity.^[^ ^40^ ^]^ However, the clinical application of these neuromodulators is often constrained by their associated side effects. Our findings suggest that MGE transplantation provides an alternative mechanism to activate the Ras‐PI3K‐PKB pathway, thereby modulating GluA1 transport and enhancing LTP expression. Unlike neuromodulators, MGE transplantation offers significant potential for future clinical applications, particularly for conditions like FXS that involve multifaceted molecular and cellular dysfunctions.

Interestingly, although Ras activation stimulates PKB signaling, pPKB levels are highly state‐dependent. The hippocampal pPKB level is significantly higher in awake mice compared to sleeping mice, underscoring its dynamic regulation compared to the relatively stable Ras‐GTP.^[^ ^37^, ^40^ ^]^ Thus, Ras‐GTP and pPKB levels do not always exhibit a direct positive correlation. To mitigate the potential confounding effects of wakefulness or sleep on pPKB dynamics, we conducted measurements at 7:00 PM, a time point reflecting an intermediate activity state. At this time, we observed increased Ras‐GTP but decreased pPKB in Fmr1‐KO mice, consistent with previous findings.^[^ ^40^ ^]^

### 3.4 Grafted INs and Atypical Neural Oscillations in Fmr1‐KO Mice

Our findings suggest that MGE transplantation may restore abnormal neural oscillations in the hippocampus of FXS mice. This restoration provides a potential pathway to evaluate the therapeutic effects of transplantation in FXS patients through electroencephalogram (EEG) analysis. Within the hippocampus, θ oscillations are critical for learning and memory encoding, while γ oscillations are involved in memory consolidation and retrieval.^[^ ^72^, ^73^ ^]^ Specifically, impairing NMDARs in PV^+^ INs results in reduced θ oscillation amplitudes, impairing spatial memory,^[^ ^74^ ^]^ while activation of PV^+^ INs induces γ oscillations, which contribute to spatial memory formation.^[^ ^75^ ^]^ These findings highlight the different roles indicate of distinct INs in regulating γ and θ oscillations.

Distinct subtypes of INs play unique roles in hippocampal rhythmic activities. PV^+^ INs regulate the phase and amplitude of θ cycles, thereby orchestrating hippocampal network oscillations.^[^ ^76^, ^77^ ^]^ SST^+^ O‐LM INs, which are phase‐locked to θ rhythms, show reduced firing rate during sharp waves.^[^ ^78^ ^]^ In contrast, nNOS^+^ ivy INs, which fire sparsely, are phase‐locked to the trough of both θ and γ oscillations.^[^ ^79^ ^]^ Our results demonstrate that MGE transplantation alters phase modulation of PN firings during hippocampal neural oscillations. It has been previously shown that tonic‐firing INs increase γ power at θ trough.^[^ ^80^ ^]^ Therefore, we hypothesize that different types of INs differentiated from MGE grafts contribute to the altered coupling between θ phases and γ oscillation amplitudes by modulating local circuits. Further investigations are required to elucidate the precise mechanisms by which newly formed INs regulate neural oscillations in the hippocampus.

## 4 Conclusion

In summary, our findings suggest that GABAergic progenitor cell transplantation could serve as a reliable therapeutic strategy for FXS, as the differentiated cells integrated effectively into the adult hippocampus. Unlike previous approaches targeting GABAR or mGluR signaling, this therapy not only corrects PN activity abnormalities but also enhances neural network oscillations by functionally integrating transplanted inhibitory neurons into existing circuits. Although generating safe and stable MGE‐like human stem cells remains a challenge, our work offers compelling evidence to constitute an important and encouraging step for cell therapy in the clinical treatment targeting FXS.

## 5 Experimental Section

### Animals

All experiments were carried out in a strict compliance with protocols (27 900) approved by the Animal Care and Use Committee at Zhejiang University School of Medicine. Mice were housed in temperature‐controlled conditions on a 12:12 h light/dark cycle with food and water ad libitum. Original Fmr1‐KO mice (stock no. 0 03205) and GFP^+^ mice (stock no. 0 06567) were obtained from the Jackson Laboratory (Bar Harbor, ME). Offspring were genotyped using PCR of genomic DNA to confirm genotypes. All experiments were performed on age‐matched male or female mice.

### Antibodies

Antibodies against FOXG1 (ab18259; RRID: AB_732 415), NeuN (ab17747; RRID: AB_443 974), AKT‐T308 (ab38449; RRID: AB_722 678), and Ki67 (ab16667; RRID: AB_302 459) were from Abcam (Cambridge, UK). Antibodies against NKX2.1 (MAB5460; RRID: AB_571 072) and SST (MAB354; RRID: AB_2 255 365) were from Millipore (Billerica, MA). The antibody against PV (pv27; RRID: AB_2 631 173) was from Swant (Burgdorf, Switzerland). The antibody against nNOS (61‐7000; RRID: AB_2 313 734) was from Thermo Fisher (Waltham, MA). Antibodies against AKT (9272; RRID: AB_329 827), Ras (67 648; RRID: AB_2 910 195) and active Ras Kit (8821) were from CST (Danvers, MA). 4′,6‐diamidino‐2‐phenylindole (DAPI; P36931; SCR_01 5961) and Alexa Fluor‐conjugated secondary antibodies (Goat anti‐rabbit Alexa Flour 488, A32731; Goat anti‐mouse Alexa Flour 488, A32723; Goat anti‐rat Alexa Flour 488, A48262; Goat anti‐rabbit Alexa Flour 594, A32740; Goat anti‐mouse Alexa Flour 594, A32742; Goat anti‐rat Alexa Flour 594, A11007) were from Invitrogen (Carlsbad, CA). Unless stated otherwise, all other chemicals were from Sigma (St. Louis, MO).

### Tissue Dissection

MGE explants were dissected from E13.5 GFP^+^ embryos in Hank's balanced salt solution (HBSS) and placed into a centrifuge tube containing pre‐cooled Leibovitz L‐15 medium. Subsequently, 2.5% Trypsin was added for digestion at 37 °C for 20 min with shaking three times every 5 min. The digestion was terminated by adding horse serum (200 µL) and DNAse (20 µL), followed by centrifugation at 1800 rpm for 6 min. After removing trypsin, the MGE tissue was gently triturated through fire‐polished Pasteur pipettes until it became a cell suspension. A high‐concentration MGE cell suspension was prepared by centrifuging for 3 min at 500 rpm at 4 °C. To verify MGE cells, the supernatant was transferred to Petri dishes containing culture media (96% Neurobasal, 2% B27, 1% GlutaMax). 2 days later, immunocytochemistry was performed in MGE cells and FOXG1^+^ and NKX2.1^+^ cells were counted using counting plates. For the control group, deactivated MGE (dMGE) was prepared by freezing at −80 °C for 2 h according to previous work,^[^ ^81^ ^]^ and cell death was confirmed by Trypan Blue staining.

### Cell Transplantation

Recipient mice (P25‐30) were anesthetized with sodium pentobarbital (0.7%; 50 mg k^−1^g) via intraperitoneal (i.p.) injection. Concentrated cell suspensions were loaded into beveled glass micropipettes (40–50 µm tip diameter) and injected (≈3 × 10^4^ cells per injection) into the hippocampus of adult Fmr1‐KO mice (P30). Using a stereotaxic apparatus (RWD Life Tech, Shenzhen, China), the injections were targeted at the DG area of the hippocampus, with coordinates set at AP −2.0 mm, ML 1.85 mm, DV 2.0 mm. Surgical sutures were used to close the skin after surgery. Successful transplantations were confirmed by assessing the density and migration of GFP^+^ cell, requiring a minimum of 30000 cells per mouse and migration ≥600 µm from the injection site in the recipient brain. Cell viability and concentration were quantified using L‐15 medium and Trypan Blue staining.

### Recombinant Virus and In Vivo Injection

AAV1‐CaMKIIα‐GluA1‐mCherry and AAV1‐CaMKIIα‐GluA1ct‐mCherry were purchased from Sunbio Medical Biotech (Shanghai, China). Original plasmid for AAV vectors with CaMKIIα promoter was obtained from Addgene (Watertown, MA). GluA1ct, the cytoplasmic termini of GluA1, was designed according to previous work.^[^ ^37^, ^42^ ^]^ The mice that had received MGE transplantation were anesthetized with sodium pentobarbital (0.7%; 50 mg k^−1^g) via i.p. injection, and the virus (100 nL) was injected into CA1 at a rate of 20 nL min^−1^ using a micro‐injector (R480, RWD Life Tech) and a micro pipette with a 10‐µm tip. The AAV injection was performed 10 days after MGE transplantation. The surgical site was closed with sutures post‐injection. Mice were allowed to recover for 20 days before undergoing electrophysiological recordings.

### Immunochemistry

Mice were anesthetized with sodium pentobarbital, decapitated, and their brains were carefully extracted. After overnight fixation at 4 °C, brains were dehydrated with 30% sucrose. Continuous frozen coronal sections (20 µm thick) spanning the hippocampus (bregma −0.95 to −4.05 mm) were obtained using a cryostat microtome (Thermo Fisher) and placed sequentially in 96‐well plates. For cell counting, slices were sequentially mounted and labeled on gelatin‐coated slides and imaged using an Olympus SLIDEVIEW VS120 (Olympus, Tokyo, Japan). For fluorescent staining, hippocampal sections were incubated in a blocking solution for 1 h at room temperature (RT). After washing with PBS, sections were incubated with primary antibodies overnight at 4 °C. Primary antibody dilutions used for immunochemistry were 1:500 (FOXG1, Ki67, NKX2.1, PV, nNOS, and SST), and 1:1000 (NeuN). After rinsing with PBS, sections were incubated with secondary antibodies (1:1000 for dilution) for 2 h at RT before staining with DAPI. Immunohistochemical images were obtained with A1R (Nikon, Tokyo, Japan) or FV1200 (Olympus) confocal microscopes. The parameters used in microscopy were consistent in all experiments.

### Preparation of Acute Hippocampal Slices

Following decapitation, mouse brains were promptly dissected and transferred to ice‐cold oxygenated sucrose‐substituted artificial cerebrospinal fluid (aCSF) consisting of (in mM) 125 sucrose, 1.25 NaH_2_PO_4_, 2 CaCl_2_, 3 KCl, 2 MgSO_4_, 26 NaHCO_3_, 11 dextrose, 1.3 sodium ascorbate and 0.6 sodium pyruvate (pH 7.30, 300 mOsm). Coronal hippocampal slices (250‐µm thick) were prepared using a vibrating tissue slicer (VT1000S, Leica, Germany) in oxygenated aCSF at 0 °C. Slices were transferred into an incubation chamber filled with the aCSF oxygenated at 95% O_2_/5% CO_2_ for 30 min (34 °C) and then for 30 min (RT) before experimentation.

### Patch‐Clamp Recording

The hippocampal slices were placed in a submerged chamber that was perfused at 2 mL min^−1^ with aCSF. Patch clamp electrodes (3–5 MΩ) were filled with an intracellular solution composed of (in mM) 134 K‐gluconate, 6 KCl, 4 NaCl, 10 HEPES, 0.2 EGTA, 4 Na_2_ATP, 0.3 Na_3_GTP, and 14 Na_2_phosphocreatine (pH 7.3, OSM 290). Neurons were visualized under an upright microscope (BX51, Olympus) equipped with a 40 × water‐immersion objective and equipped with infrared differential interference contrast optics. Whole‐cell patch‐clamp recordings were obtained with an Axon MultiClamp 700B amplifier (Molecular Devices, CA). Currents were digitized at 10 kHz and filtered at 3 kHz. Offline analyses were conducted using a sliding template algorithm (ClampFit 10, Molecular Devices). mEPSCs and mIPSCs were recorded in whole‐cell configuration in the presence of 0.5 µM tetrodotoxin (TTX) plus GABAzine (10 µM) or NBQX, respectively. The offline analysis of mEPSCs and mIPSCs was conducted using ClampFit 10. To record AMPA and NMDA responses, a bipolar electrode was placed in the stratum radiatum (200 µm away from recorded PN), and synaptic responses were evoked by single voltage pulses (200 µs). Synaptic AMPA responses at −60 and +40 mV were averaged from 10 trials, and their ratio was used as an index of rectification. The firings of fast‐spiking and regular‐spiking INs were kept constant during recording. Moreover, the firing frequency was typically 70–100 Hz for fast‐spiking INs or 20–40 Hz for regular‐spiking INs. In contrast, burst‐spiking INs typically displayed irregular and bursting firing patterns.

### Extracellular Recording

Hippocampal fEPSPs were evoked at 0.05 Hz using a bipolar concentric electrode positioned in the CA3 region and recorded with an aCSF‐filled pipette (1–2 MΩ) placed in the SC of CA1. Stimulation of SC inputs was achieved using monophasic pulses. LTP was induced by θ‐busrt stimulation (TBS), which comprised 4 1‐s trains of 100 Hz pulses, delivered at 20‐s intervals, with the stimulus intensity set at 20%–30% of the spike threshold. The fEPSPs were analyzed using ClampFit 10.

*scRT‐PCR*: Cytoplasm from individual INs was obtained after 10‐min of whole‐cell recording, followed by 1‐min of negative pressure applied via mouth suction. Harvested mRNA was first reverse‐transcribed to cDNA using the Super Script III kit (Invitrogen), incubated at 50 °C for 50 min, followed by termination at 65 °C for 20 min. The cDNA was amplified using multiplex PCR (25 cycles) with the QIAGEN Multiplex PCR Master Mix kit (Qiagen, Germany) in a 100 µL reaction volume. The transcripts of each tested gene were detected by a second round of PCR using an individual primer set: 1 µL of multiplex PCR mixture was applied as a template in a 10‐µL reaction with HotStarTaq polymerase (TianGen, China), amplified for 35 cycles. PCR products were run on a 2% agarose gel and visualized under ultraviolet light. Primer sequences for amplification were as follows: *GFP, F: 5′‐CGA CGG CAA CTA CAA GAC CC‐3′; R: 5′‐ GGT AGT GGT TGT CGG GCA GC ‐3′. PV, F: 5′‐ CGG ATG AGG TGA AGA AGG TGT ‐3′; R: 5′‐ TCC CCA TCC TTG TCT CCA GC ‐3′. SST, F: 5′‐ GCA TCG TCC TGG CTT TGG G ‐3′; R: 5′‐ GGG CTC CAG GGC ATC ATT CT ‐3′. nNOS, F: 5′‐ CCT GTC CCT TTA GTG GCT GGT A ‐3′; R: 5′‐ GAT GAA GGA CTC GGT GGC AGA ‐3′. Lhx6, F: 5′‐ GAC GAA GGT AGA GCC TCC CCA TGT ‐3′; R: 5′‐ TGC CTC AGC GAT GTG CGA CAC A ‐3′*.

### Open Field Test (OFT)

For all behavioral tests, the mice were extensively handled daily for 3 days before experiments, and 75% ethanol was used to clean behavioral chambers between trials. For the OFT, mice were placed in a novel, brightly‐lit square (46 × 46 × 28 cm, length × width × height) plexiglass chamber for 15 min. The activity of mice was recorded and analyzed using Any‐maze software (Stoelting, IL). During analysis, the arena was subdivided into 2 concentric zones named inner (23 × 23 cm, length × width) and outer zones, respectively. Time spent and travel distance of animals in each zone were recorded.

### Three Chamber Test

The apparatus consisted of a rectangular plexiglass box (60 × 35 × 10 cm, length × width × height) evenly divided into three chambers. The test mouse was initially placed in the central chamber for a 10‐min habituation. During the first examination of social behavior, a stimulus mouse (S1) was introduced into a wire cage in one chamber, while the opposite chamber remained empty to serve as an inanimate object with no social valence. The test mouse was allowed to freely explore all three chambers over 10 min. Next, the S2 mouse was introduced into the previously empty chamber, replacing the inanimate object. As a second examination, the test mice were again allowed to spend 10 min to explore all 3 chambers. The time spent in each chamber was recorded, and for the first examination the ratio of (S1‐E) to (S1+E) was calculated as the preference index (S1‐E), and for the second examination the ratio of (S2‐S1) to (S2+S1) was measured as the preference index (S2‐S1).

### T‐maze Test

Mice were placed at the base of a T‐maze, which consisted of 2 arms (50 cm in length), and allowed to freely explore either arm over 10 consecutive trials. A choice was counted when the mouse stepped into one arm with all 4 paws. Upon selecting an arm, the gate to that arm was immediately closed, and the mouse was allowed to explore there for 5 s. The mouse was then gently returned to the starting point for the next trial. If the mouse selected the same arm in 2 consecutive trials, this was scored as one repeat.

### Grooming Test

Spray‐induced grooming was performed according to previous work.^[^ ^82^ ^]^ Each mouse was individually placed in an empty standard cage (45 × 45 × 50 cm) to prevent digging behavior and allow a 5 min habituation period. Subsequently, the mouse was misted from above with water (23 °C) using a manual spray device. The spray adequately coated the dorsal surface of the mouse with a fine mist. Mouse activity was then recorded for 10 min with a high‐speed camera (30 frames/s) and analyzed by a trained observer. Cumulative time and bouts of grooming were scored from the recorded videos.

### Marble Burying Test

A clean cage (50 × 50 × 18 cm) was filled with 4‐cm‐depth bedding material and 25 glass marbles. The marbles were arranged in an equidistant 5 × 5 grid. Animals were given access to the marbles for 30 min. Afterward, the burying of marbles was scored. The score was 1 when the marble was 100% covered or 0.5 when it was just partially covered. All scoring was performed by 2 independent researchers to ensure reliability.

### Morris Water Maze (MWM)

The MWM consisted of a large circular tank (1 m in diameter, 0.5 m high) of water maintained at 25 °C and made opaque by the addition of nontoxic water‐based white paint. An escape platform (11 cm in diameter) was submerged 0.5–1 cm below the surface and placed at a fixed position in the center of one quadrant for all trials. Visual cues were placed on the walls of the testing room to serve as spatial references. An automated tracking system (DigBehv‐MWM; Jiliang Software Tech, Shanghai, China) monitored performance using the following parameters: escape latency (finding the submerged platform), swimming speed, and a visual sensitivity test.

### Novel Object Recognition

Mice were first introduced to a rectangular apparatus (50 × 50 × 30 cm) for 10 min of free exploration. After 30 min, 2 cylindrical blocks were placed in the opposite corners of the apparatus, and the mouse was reintroduced for an additional 10 min of exploration. Finally, one of the cylindrical blocks was replaced with a square‐shaped block at its original location. The mouse was reintroduced into this apparatus 30 min later for a 10‐min exploration. The interaction of the mouse with cylindrical and square‐shaped blocks was video‐recorded at this stage using the Anymaze software.

### Novel Place Recognition

First, one mouse was introduced to a rectangular apparatus (50 × 50 × 30 cm) where it could freely explore for 10 min. Subsequently, 2 cylindrical blocks were placed in the opposite corners of the apparatus, and the mouse was re‐introduced into this environment 30 min later for free exploration over 10 min. Finally, one of the cylindrical blocks was relocated from its original position to a new location. The mouse was reintroduced 30 min later for another 10‐min exploration session. Mouse interactions with the 2 cylindrical blocks were video‐recorded and analyzed using Anymaze software.

### Conditioning Fear Test

On day one, mice were placed in a conditioned fear box and allowed to freely explore for 2 min. Subsequently, they were exposed to 20‐s tone stimulation at 80 decibels, immediately followed by 1‐s electrical foot shock. This sequence was repeated 5 times, after which the mice remained in the box for an additional 2 min. On day two, the mice were reintroduced into the box for another 2‐min session, during which their freezing duration was recorded as a measure of context fear conditioning (CFC). On day three, the mice were placed in a novel box with striped patterning on its inner wall and subjected to a 20‐s tone at 80 decibels. Their freezing durations during the subsequent 2 min were recorded to assess tone fear condition (TFC).

### Rotarod Test

After habituation on a rotarod rotating at 5 rpm, three groups of female mice were tested twice daily for 4 consecutive days. During each session, the rotarod velocity increased with a constant acceleration of 9 rpm min^−2^, starting at 5 rpm and reaching up to 50 rpm.

### Western Blotting

Protein concentrations were determined using a BCA protein assay. Equal quantities of proteins were loaded and fractionated on SDS‐PAGE and transferred to the PVDF membrane (Immobilon‐P, Millipore). The membranes were immunoblotted with antibodies and visualized by enhanced chemiluminescence (Thermo). The dilutions of primary antibodies were 1:1000 for Ras; 1:5000 for p‐PKG; 1:10000 for HA, His, Flag, GAPDH, and PKCα. Secondary antibodies were goat anti‐rabbit (1:10000), and goat anti‐mouse (1:10000). Film signals were digitally scanned and quantified using ImageJ 1.42q (NIH, Bethesda, MD).

### TUNEL Assay

A kit (Roche, Palo Alto, CA) was used for the Terminal deoxynucleotidyl transferase (TdT)‐mediated dUTP nick end labeling (TUNEL) assay. Tissues were fixed in 4% paraformaldehyde and 4% sucrose for 15 min and processed for antigen retrieval with 0.1% Triton X‐100 and 0.1% sodium citrate for 2 min on ice. The tissues were subsequently incubated with a TUNEL reaction mixture containing TdT in a humidified 37 °C chamber for 1 h.

### In Vivo Recording

Mice were anesthetized with sodium pentobarbital (50 mg k^−1^g, i.p.) and secured on a stereotaxic apparatus (RWD Life Tech, Shenzhen, China). A heating pad maintained body temperature at ≈37 °C. After shaving the hair and incising the scalp, the connective tissue was gently removed with cotton swabs. A microelectrode array (4 × 4; Kedou Tech, Suzhou, China) was lowered into the hippocampal CA1 (AP: −2.00 mm; ML: +1.30 mm) to record local electrical signals. A craniotomy hole (AP: 2.00 mm; ML: −0.50 mm) was drilled, and a miniature stainless screw was inserted into the hole as the ground. Electrical signals were amplified and sampled at 30K Hz using NeuroStudio V3.3 software (Greathink Med Tech, Nanjing, China). The electrical signals were recorded while the recording electrode slowly descended, and the electrodes were affixed to the skull with dental cement when all channels recorded spike signals. After electrode implantation, mice were allowed to recover in the residential chamber, and in vivo recording was performed 5 days later.

### Neuronal Classification

Spike waveform (width and asymmetry), autocorrelation properties, and firing rates were used to classify putative PNs and INs. Autocorrelation was calculated using a double exponential model according to previous work.^[^ ^59^ ^]^

(1) $$ACG_{base}=\left(\beta_{1}+\beta_{2}\right)\times e^{-\frac{x^{2}}{\beta_{3}}}$$

for x ≤ 0 otherwise *ACG_base_* = 0

(2) $$ACG_{burst}=\beta_{1}\times e^{-\frac{x^{\mathrm{round}\left(\beta_{4}\right)}}{\beta_{5}}}+\beta_{2}$$

for x > 0 otherwise *ACG_burst_* = 0. Where 0.9 < *β*_4_ < 2.1.

(3) $$ACG=ACG_{base}+ACG_{burst}$$

### Spike Sorting

The raw signals were imported into LabChart (A‐M Systems, Sequim, WA) for high‐pass filtering, and the signals with frequencies below 250 Hz were analyzed. Subsequently, threshold detection combined with principal component analysis from SpikeSort 3D software (NeuraLynx, Bozeman, MT) was employed to automatically transfer spike signals to 2D partitions. The resulting spikes were grouped into specified units and then imported into LabChart for further analysis of firing frequency, inter‐spike interval (ISI), and autocorrelation to determine whether they represent INs or PNs.

### LFP Analysis

The spectrum function embedded in LabChart software was employed to analyze raw signals with frequencies less than 250 Hz, enabling the extraction of power values for each firing band. Subsequently, cumulative power values for θ, low γ, and high γ bands are computed along with their respective proportions relative to the total power.

### Spike‐Count Correlation Analysis

Spike rates of individual neurons were calculated in 100 ms intervals over a time range of 100–1000 ms. The average firing rates across multiple mice were computed along with their standard deviations. rSC, the spike‐count correlation between 2 neurons, was calculated according to Equation (4).

(4) $$\mathrm{rSC}=\sum_{i=1}^{n}\left(\begin{array}{c} n\\ i \end{array}\right)\left(X^{i}-\mu X\right)\;*\;\left(Y^{i}-\mu Y\right)\;/\left(\sigma X\;*\;\sigma Y\right)$$

where *X^i^* and *Y^i^* are firing rate of neurons *X* and *Y* during a unit time for experiment *i*; *μX* and *μY* are the averages of firing rates of neurons *X* and *Y*; *σX* and *σY* indicate the standard deviations of firing rates for neurons *X* and *Y* during a unit time.

### Spike‐Phase Locking Analysis

LFPs (1–250 Hz) of PNs were segmented into 50 frequency bands logarithmically. The phases of each band were discretized into 36 units spanning from ‐π to π using Matlab software. Subsequently, the occurrences of PN spiking at different phases across all LFP frequency bands were tallied to compute the Rayleigh's Z values according to Equation (5).

(5) $$\begin{array}{c} X=\sum_{I=1}^{N}\left(\cos\;a_{i}\right)/N\\ Y=\sum_{i=1}^{\infty }\left(\sin\;a_{i}\right)/N\\ r=\sqrt{X^{2}+Y^{2}}\\ Z=Nr^{2} \end{array}$$

where *N* is the total number of PN spikes within a specific frequency band; *α*_i_ is the phase of the individual spike. The rose diagrams depicting spike distribution across different phases of PNs in various frequency bands are generated using Matlab.

### Phase‐Amplitude Coupling (PAC) Analysis

PAC describes how the amplitude of γ oscillations is modulated by the phase of a θ signal. The results of PAC are shown in a comodulogram with the x‐axis as the phase of oscillations at θ frequencies and the y‐axis as the amplitude of oscillations at γ frequencies. LFP signals (5–200 Hz) were segmented with a step size of 5 Hz and a bandwidth of 4 Hz, resulting in a set comprising 40 *X*_A_(*t*) from 40 bands. Similarly, LFP signals (2–20 Hz) were divided using a step size of 1 Hz and a bandwidth of 1 Hz, generating another set X_P_(*t*) consisting of 19 frequency bands. Next, the amplitude *A*_γ_(*t*) from *X*_A_(*t*) and the phase *θ*_θ_(*t*) from X_P_(*t*) were extracted respectively to generate a new set *Z*(*t*) using Equation (6).

(6) $$Z\left(t\right)=A_{\gamma }\left(t\right)*\exp\left[i*\theta_{\theta }\left(t\right)]=\mathrm{real}[Z\left(t\right)]+\mathrm{imag}[Z\left(t\right)\right]$$

Then the set *Z*(*t*) is normalized to yield *MI*_raw_ according to Equation (7).

(7) $$MI_{\mathrm{raw}}=\mathrm{ABS}\left(\mathrm{MEAN}(Z\left(t\right)\right)$$

Then, *A*_γ_(*t*) and *θ*_θ_(*t*) are aligned 200 times through misalignment, followed by the aforementioned mathematical analysis to obtain the value of 200*MI*_raw_. Next, a Gaussian fit is applied to 200*M*_raw_ to derive the mean value μ and variance value σ of Gaussian distribution through a best‐fit process. Subsequently, *MI* is computed using Equation (8).

(8) $$MI=\left(MI_{\mathrm{raw}}-\mu \right)/\sigma$$

*MI* indicates the relationship between θ wave phases and γ wave amplitudes.

### Statistical Analysis

All experiments and data analyses were conducted with the experimenters blinded to the genotypes until data integration. Statistical analyses were performed using Igor Pro 6.0 (Wavemetrics, Lake Oswego, OR), GraphPad Prism 8.0, SPSS 17.0 (IBM, Chicago, IL), MatLab (Zhejiang University version), and Rx64. Statistical differences were determined using unpaired two‐sided Student's *t*‐test for two‐group comparison or ANOVA followed by LSD's post hoc test for multiple comparisons. In particular, the results in Figure 4B and Figure S8I (Supporting Information) were analyzed using repeated measures ANOVA; the statistics in Figure 5B,C and Figure S10F (Supporting Information) were conducted using two‐way ANOVA followed by LSD's post hoc test. The level of significance was set at *p* < 0.05. *n* represents the number of animals, cells, or batches depending on experimental design. Data in the text and figures are presented as the mean ± SEM.

## Conflict of Interest

The authors declare no conflict of interest.

## Author Contributions

C.W., J.‐Y.L., L.‐D.S., and X.‐T.W. contributed equally to this work. C.W., L.D.S., L.W., and Y.S. conceived and designed the experiments. C.W. and X.T.W. performed biochemistry and behavioral experiments. C.W., J.Y.L., Y.P.B., Z.X.W., and L.Y.Y conducted electrophysiological experiments. C.W., J.Y.L., L.D.S., Y.P.B., X.T.W., Z.X.W., M.L., X.J.L., L.Z., W.C., W.Y., J.L., L.W., and Y.S. analyzed and interpreted results. C.W., L.D.S., J.L., L.W., and Y.S. wrote the manuscript.

## Supporting information



Supporting Information

## Data Availability

The data that support the findings of this study are available from the corresponding author upon reasonable request.
