# Supplementary material for: GABAergic Progenitor Cell Graft Rescues Cognitive Deficits in Fragile X Syndrome Mice
Source: Adv Sci (Weinh). 2025 Jan 17;12(10):2411972. doi: 10.1002/advs.202411972 (PMC11904963; doi:10.1002/advs.202411972)
Supplement: Supplementary file 1 — Supporting Information [file ADVS-12-2411972-s001.docx]

**
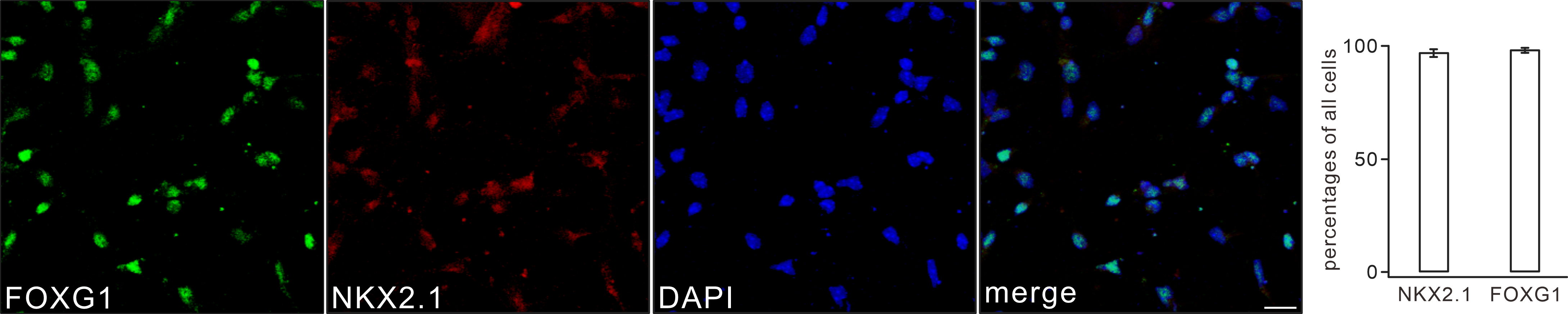
**

**Figure S1**. Immunofluorescent staining of FOXG1, NKX2.1 and DAPI in cultured MGE cells. Scale bar: 20 μm. Bar graphs indicate the proportions of FOXG1^+^ (98.5 ± 1.1%; *n* = 8 mice) and NKX2.1^+^ (97.1 ± 1.8%; *n* = 8 mice) cells.

**
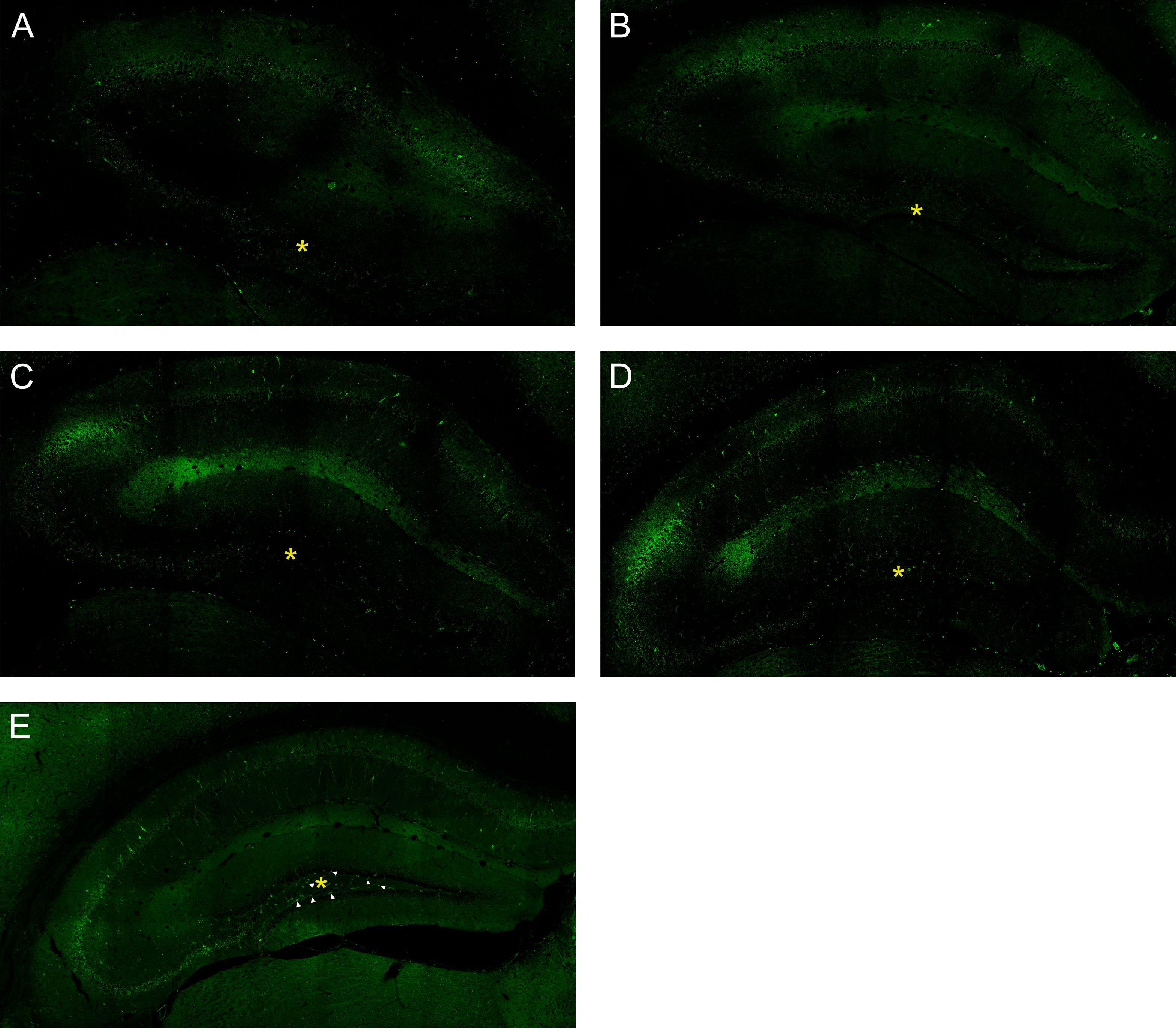
**

**Figure S2**. A-E) show uncompressed images in Figure 1C (from left to right). In panel E, the arrowheads indicate example GFP^+^ MGE cells nearby the injection site (asterisk).

**
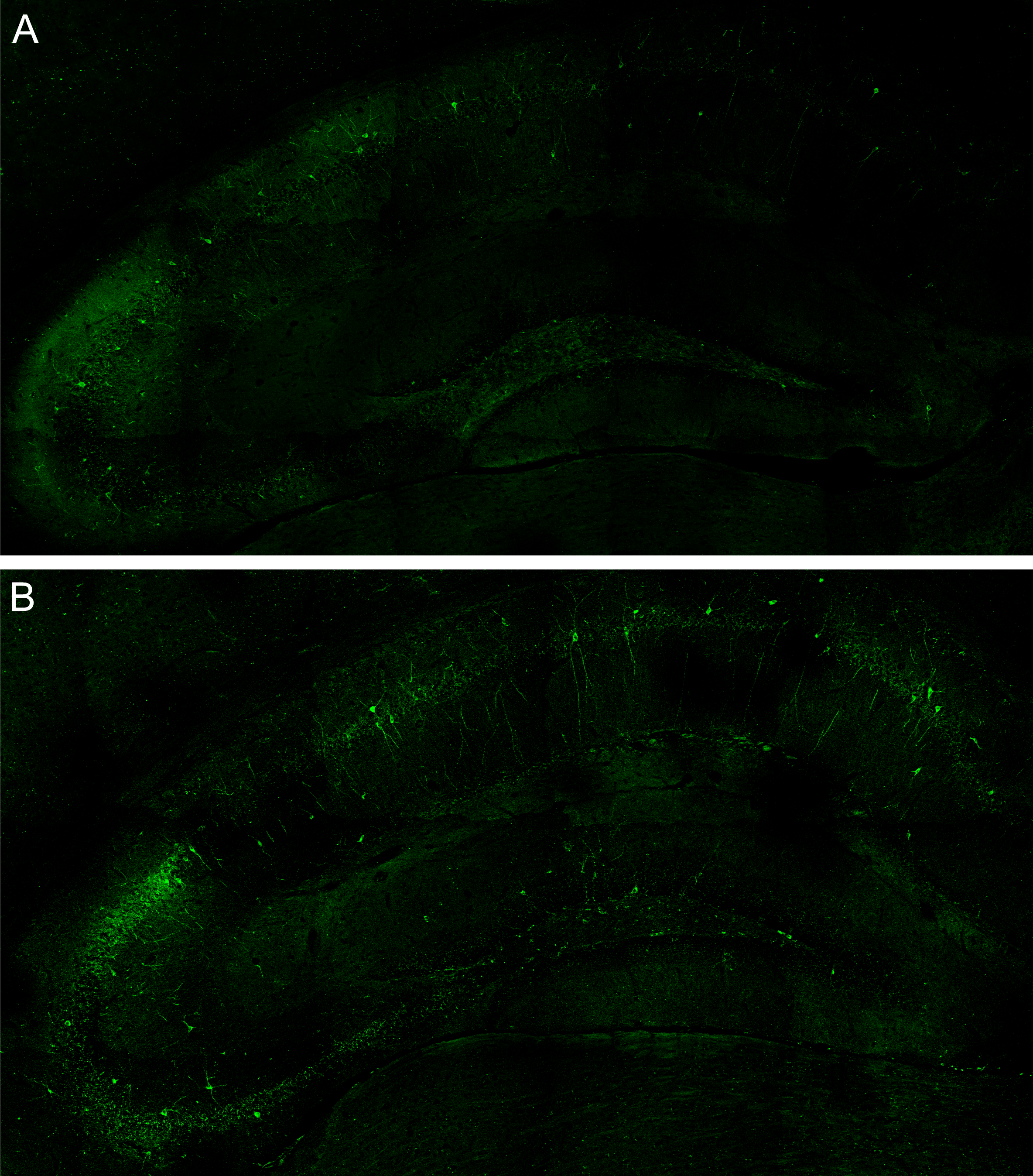
**

**Figure S3**. Uncompressed images in Figure 1D. A) For upper panel of Figure 1D (7 DAT). B) For lower panel of Figure 1D (60 DAT).

**
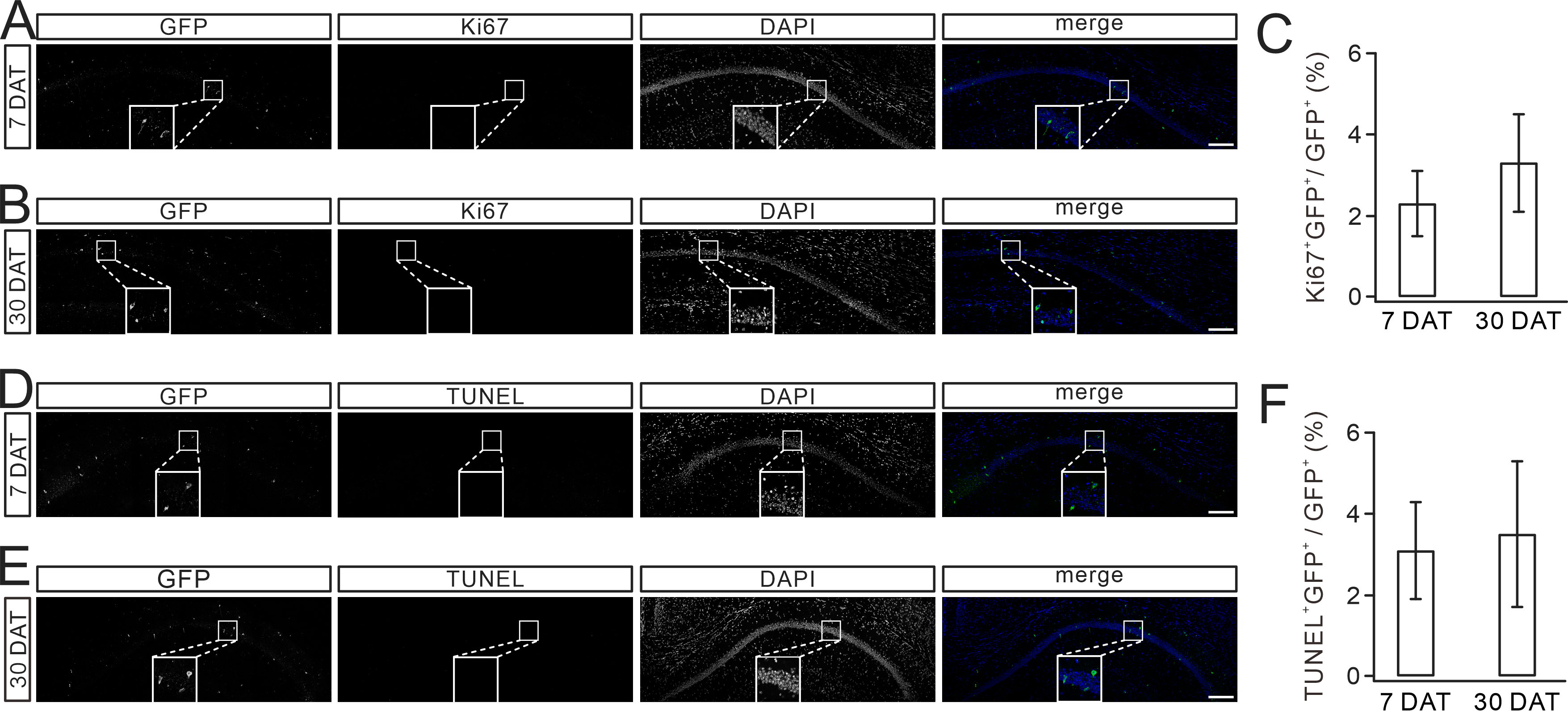
**

**Figure S4**. No difference in MGE cell proliferation and survival ratios between 7 DAT and 30 DAT. A) Example images of MGE cells co-labeled with Ki67 and DAPI at 7 DAT. Scale bar: 100 μm. B) Example images of MGE cells co-labeled with Ki67 and DAPI at 30 DAT. Scale bar: 100 μm. C) The proportions of Ki67^+^ cells in GFP^+^ MGE cells. 7 DAT: 2.4 ± 0.8%. 30 DAT: 3.3 ± 1.2%. *p* = 0.14. *n* = 4 mice per group. D) Example images of MGE cells co-staining of TUNEL and DAPI at 7 DAT. Scale bar: 100 μm. E) Example images of MGE cells co-staining of TUNEL and DAPI at 30 DAT. Scale bar: 100 μm. D) The proportions of TUNEL^+^ cells in GFP^+^ MGE cells. 7 DAT: 3.1 ± 1.2%. 30 DAT: 3.5 ± 1.8%. *p* = 0.37. *n* = 4 mice per group.

**
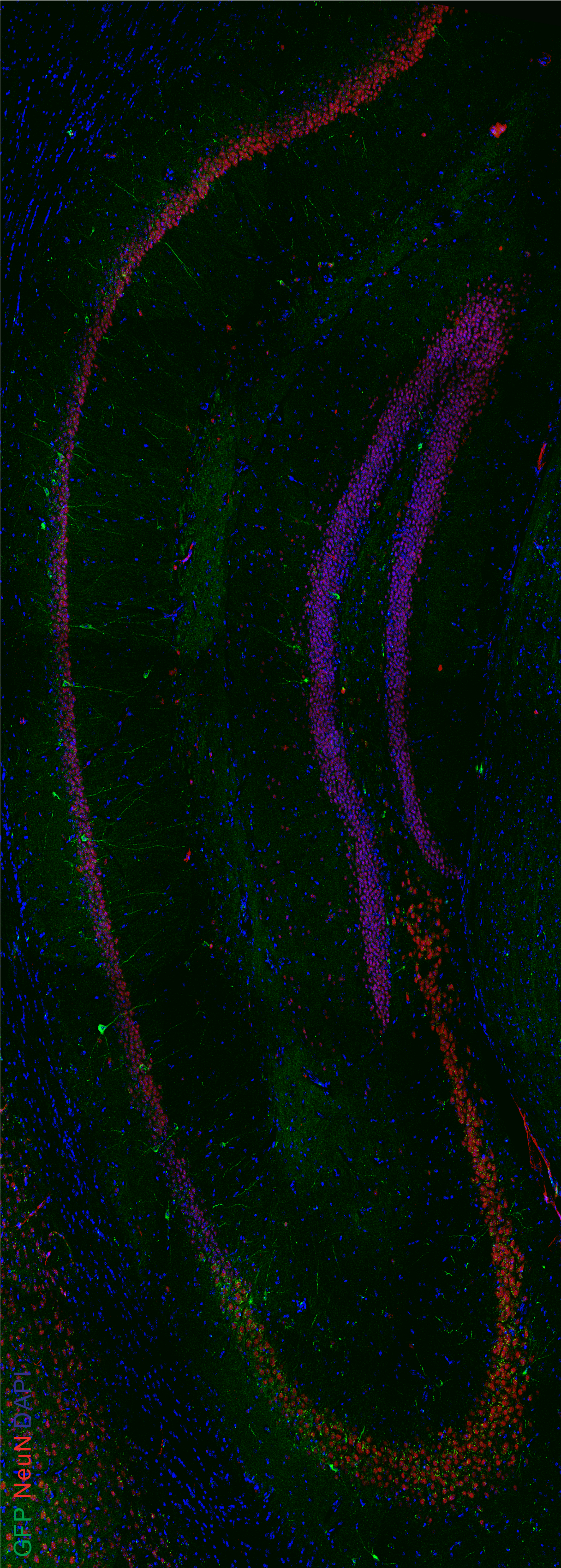
**

**Figure S5**. The uncompressed image in Figure 1F, showing GFP, NeuN and DAPI fluorescence.

**
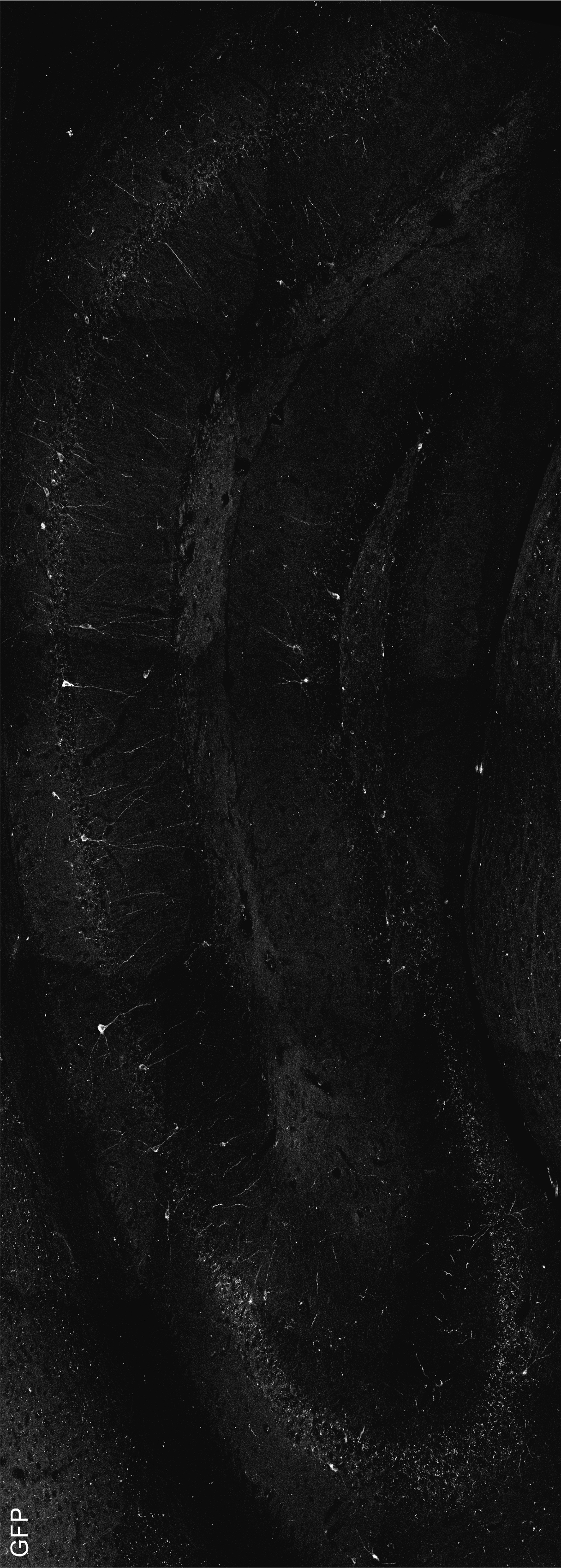
**

**Figure S6**. The uncompressed image in Figure 1F, showing GFP fluorescence.

**
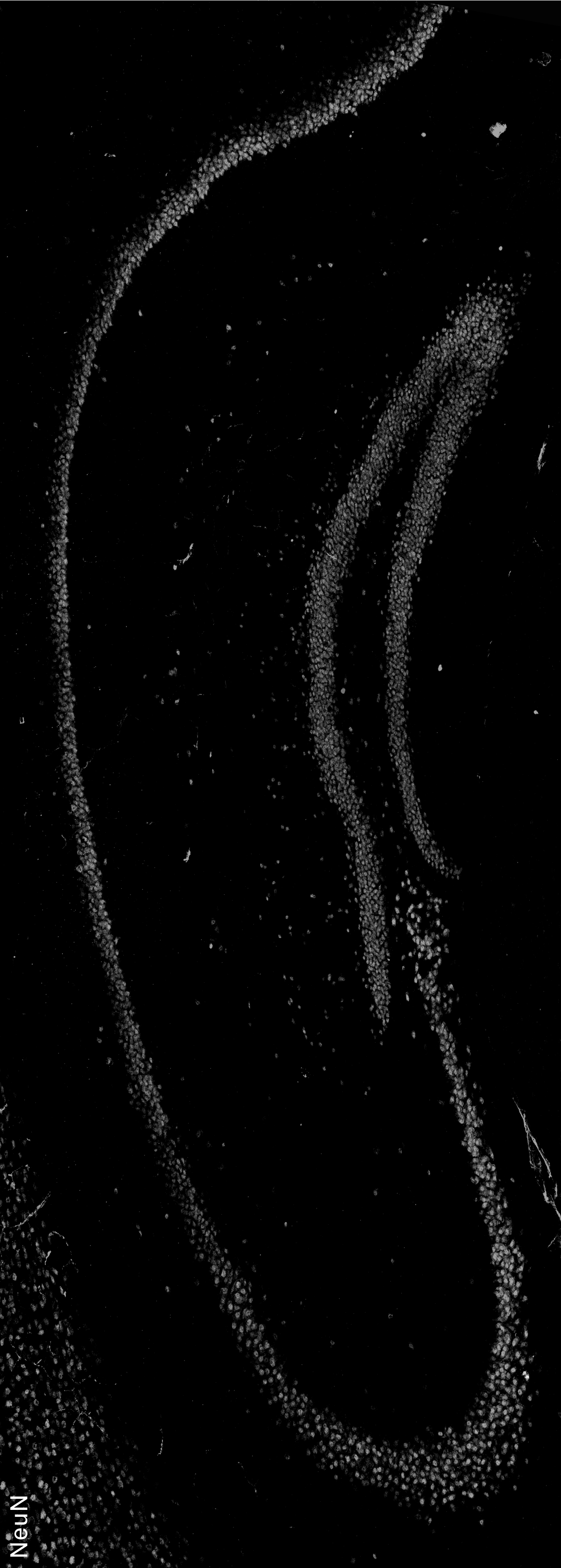
**

**Figure S7**. The uncompressed image in Figure 1F, showing NeuN fluorescence.

**
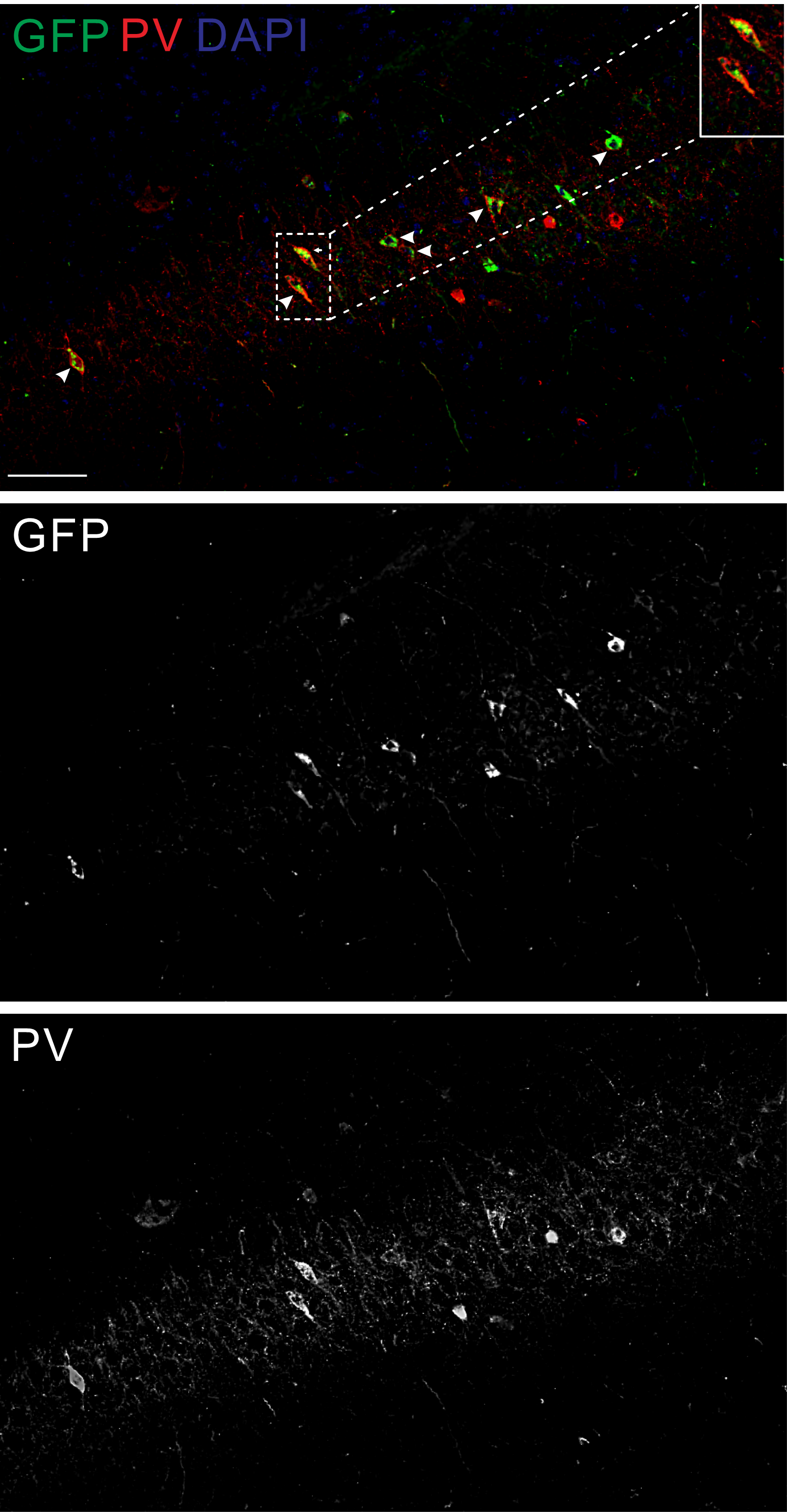
**

**Figure S8**. Uncompressed images in Figure 1G, showing GFP, PV and DAPI fluorescence in combined or single color.

**
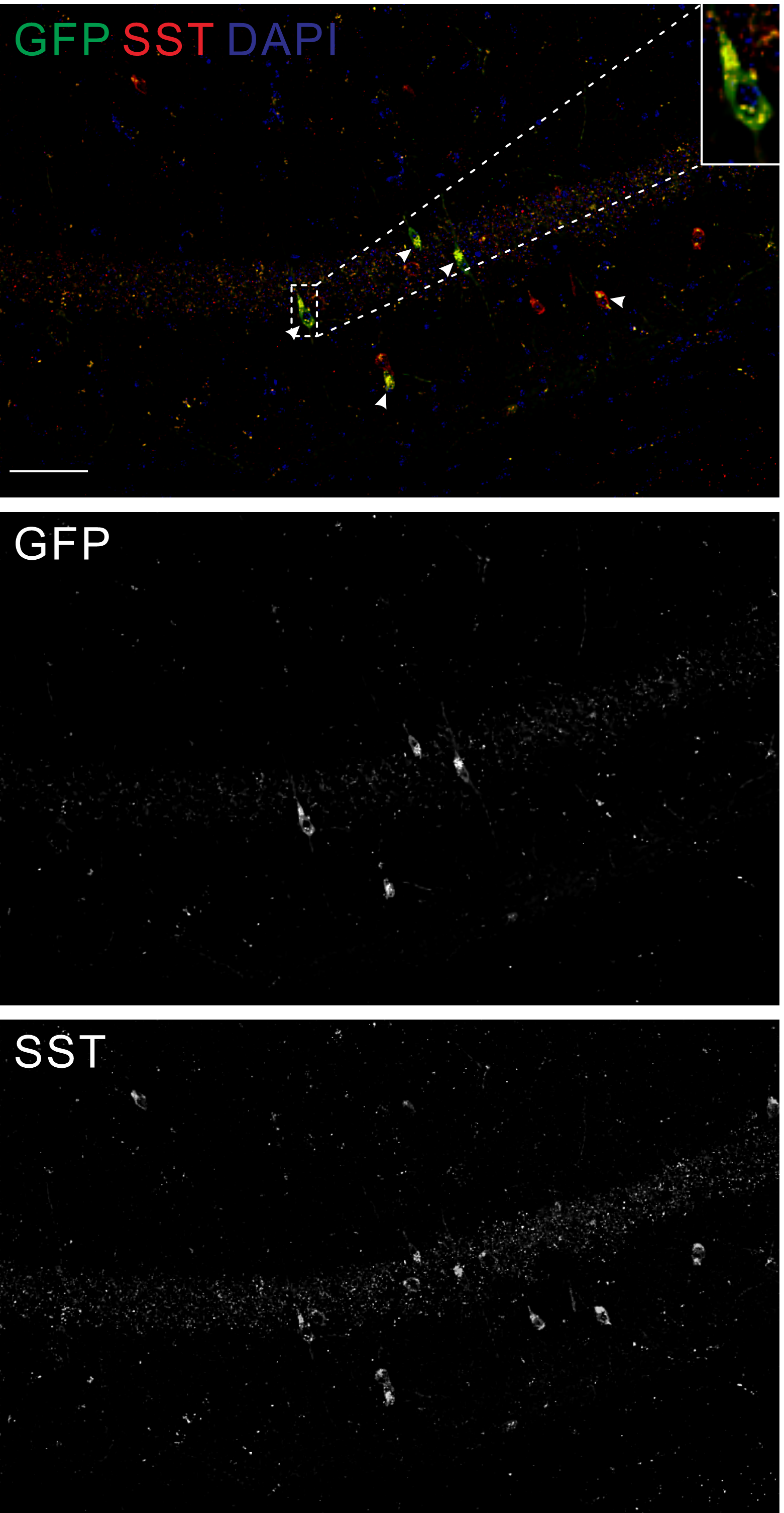
**

**Figure S9**. Uncompressed images in Figure 1G, showing GFP, SST and DAPI fluorescence in combined or single color.

**
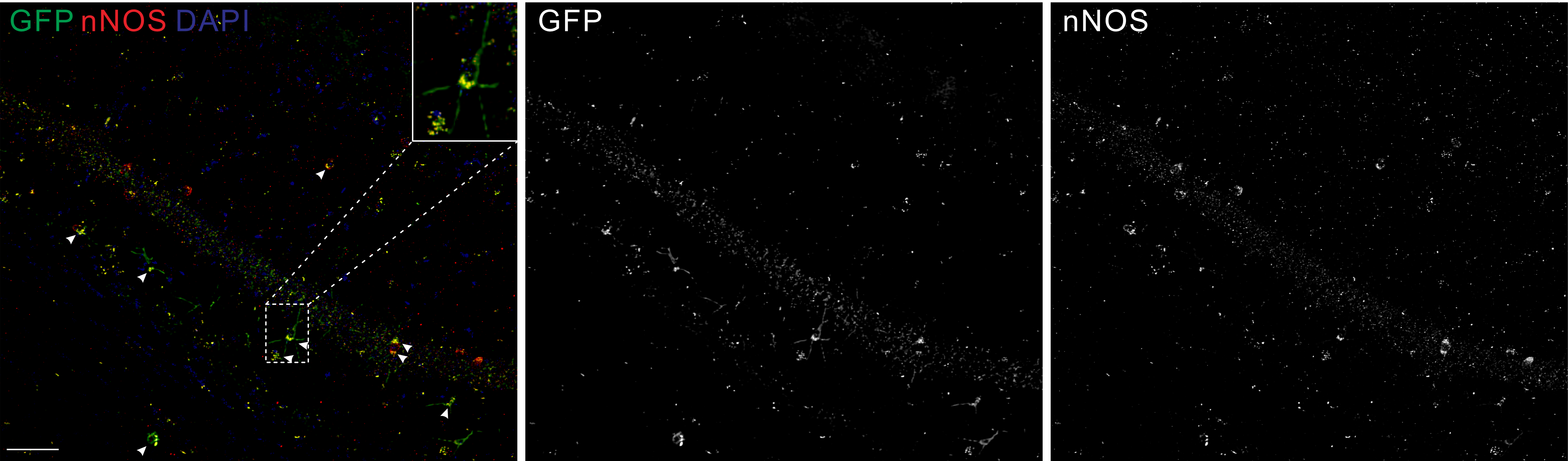
**

**Figure S10**. Uncompressed images in Figure 1G, showing GFP, nNOS and DAPI fluorescence in combined or single color.

**
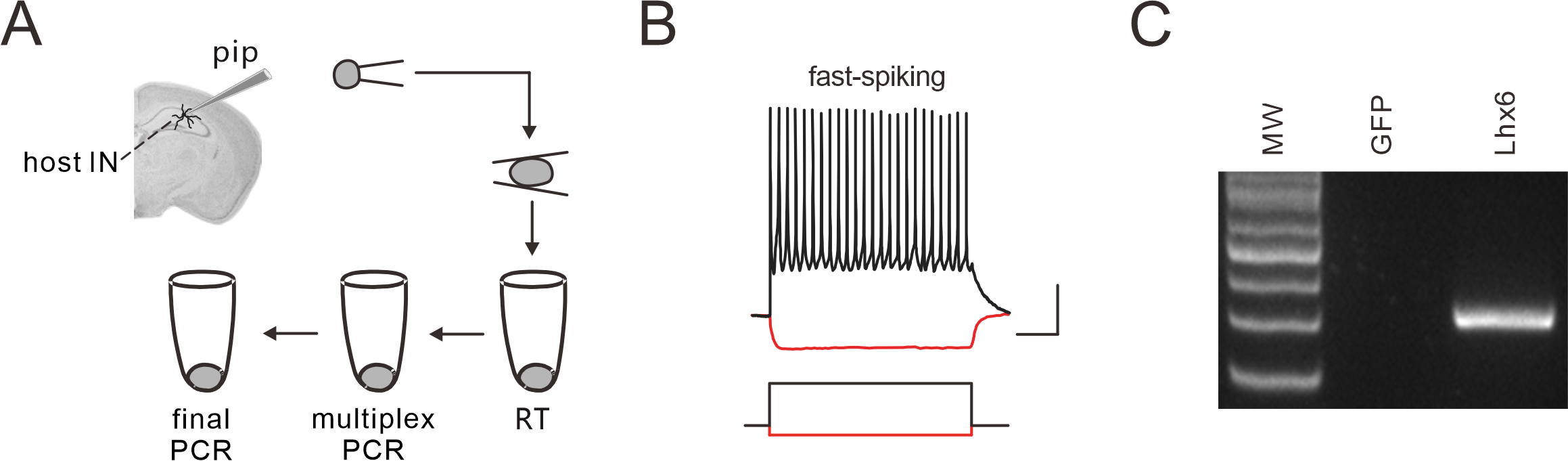
**

**Figure S11**. Validation of scRT-PCR. A) Schematic showing scRT-PCR in a host IN. B) Example electrophysiological response of a host IN to injected currents from a holding potential near −70 mV. Scale bars: 20 mV / 200 ms. C) RNA profiles obtained from the host IN.

**
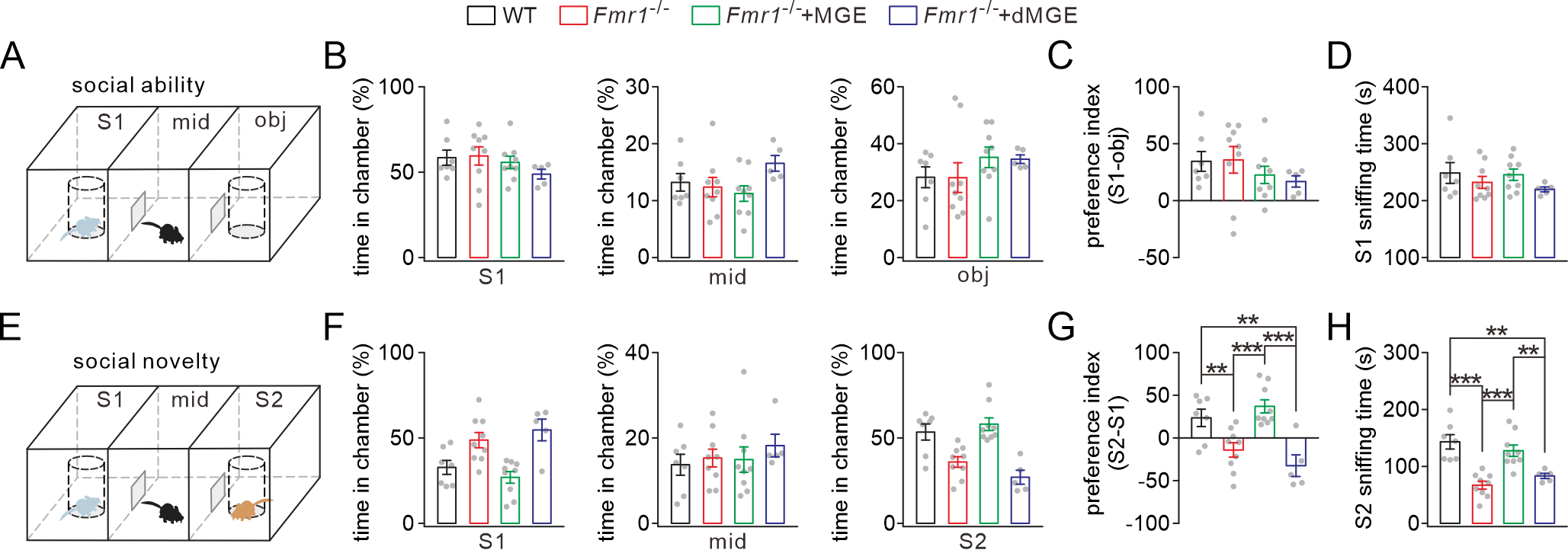
**

**Figure S12**. Performances of four groups of mice in three-chamber test. Note that the ages were P60 for WT and Fmr1-KO mice, but 30 DAT for Fmr1-KO+MGE and Fmr1-KO+dMGE mice. A) Schematic diagram illustrating social ability detection in three chamber test. B) Percentages of time spent of mice in S1, middle (mid) box and object (obj) box. C) Preference index (S1-obj) / (S1+obj) of mice. D) Sniffing time with S1. E) Schematic diagram illustrating social novelty detection. F) Percentages of time spent of mice in S1, middle (mid) box and S2. G) Preference index (S2-S1) / (S1+S2) of mice. H) Sniffing time with S2. See Table S4 (Supporting Information) for statistics, including *n* values, *p* values, and specific statistical tests. ***p* < 0.01. ****p* < 0.001.

**
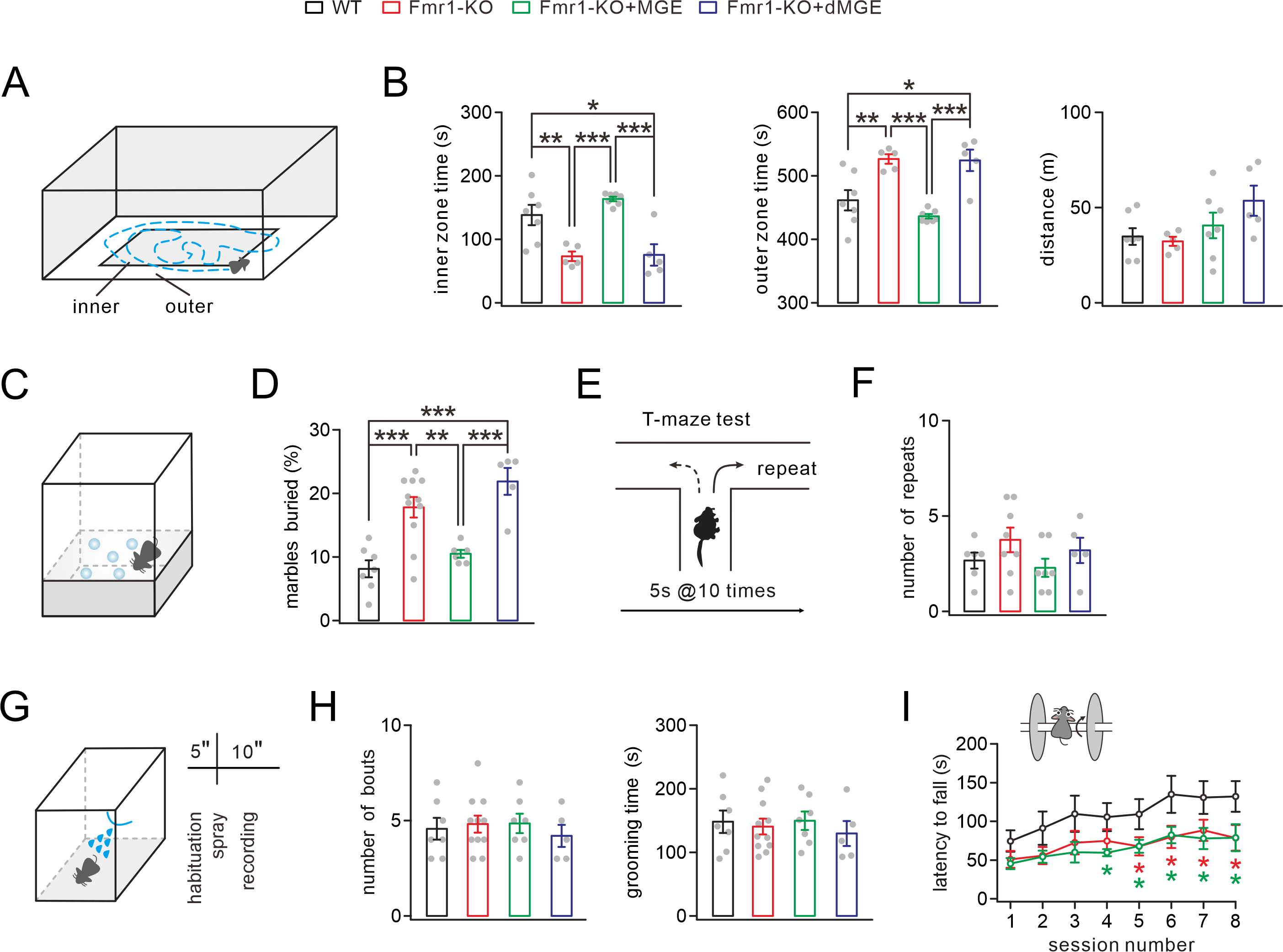
**

**Figure S13.** Performances of four groups of mice in OFT, marble burying, grooming, and rotarod tests. Note that the ages were P60 for WT and Fmr1-KO mice, but 30 DAT for Fmr1-KO+MGE and Fmr1-KO+dMGE mice. A) Schematic showing OFT. B) Statistics of time spent in the inner space, outer space and total walking duration. C) Schematic showing marble burying test. D) Statistics of buried beads. E) Schematic showing T-maze test. F) Statistics of number of repetitions. G) Schematic showing grooming test. H) Statistics of number of grooming bouts and duration. I) Time spent on the rotarod. Green and red asterisks indicate the difference of Fmr1-KO or Fmr1-KO+MGE *vs* WT. See Table S5 (Supporting Information) for statistics, including *n* values, *p* values, and specific statistical tests. **p* < 0.05. ***p* < 0.01. ****p* < 0.001.

**
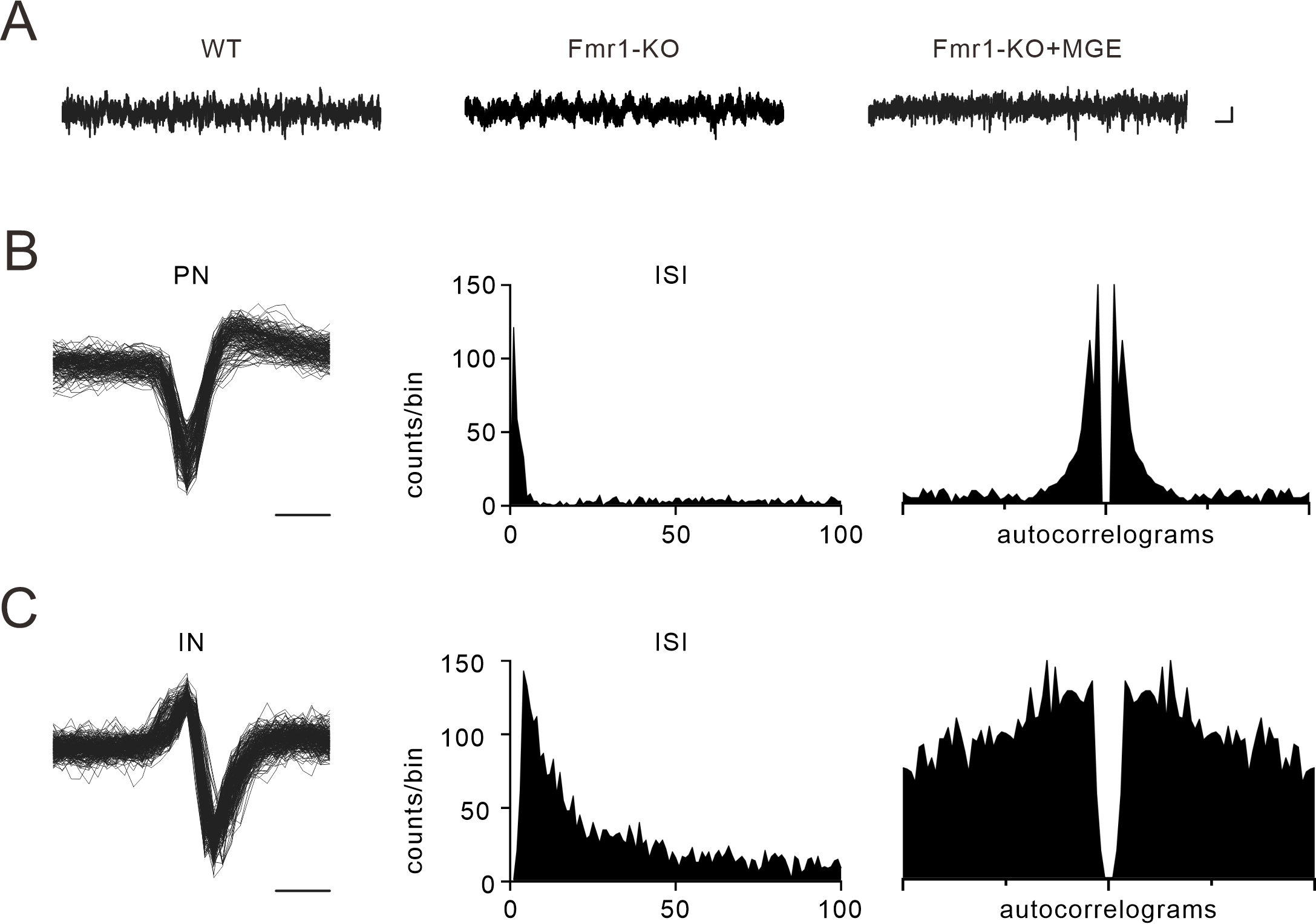
**

**Figure S14.** Firings of PNs and INs *in vivo*. A) Example raw LFP traces from WT, Fmr1-KO and Fmr1-KO+MGE mice. Scale bars: 4 s / 50 μV. B) Left panel: Example firings of PNs. Middle panel: firing intervals. Right panel: autocorrelation analysis of PNs. C) Left panel: Example firings of INs. Middle panel: firing intervals. Right panel: autocorrelation analysis of INs.

**
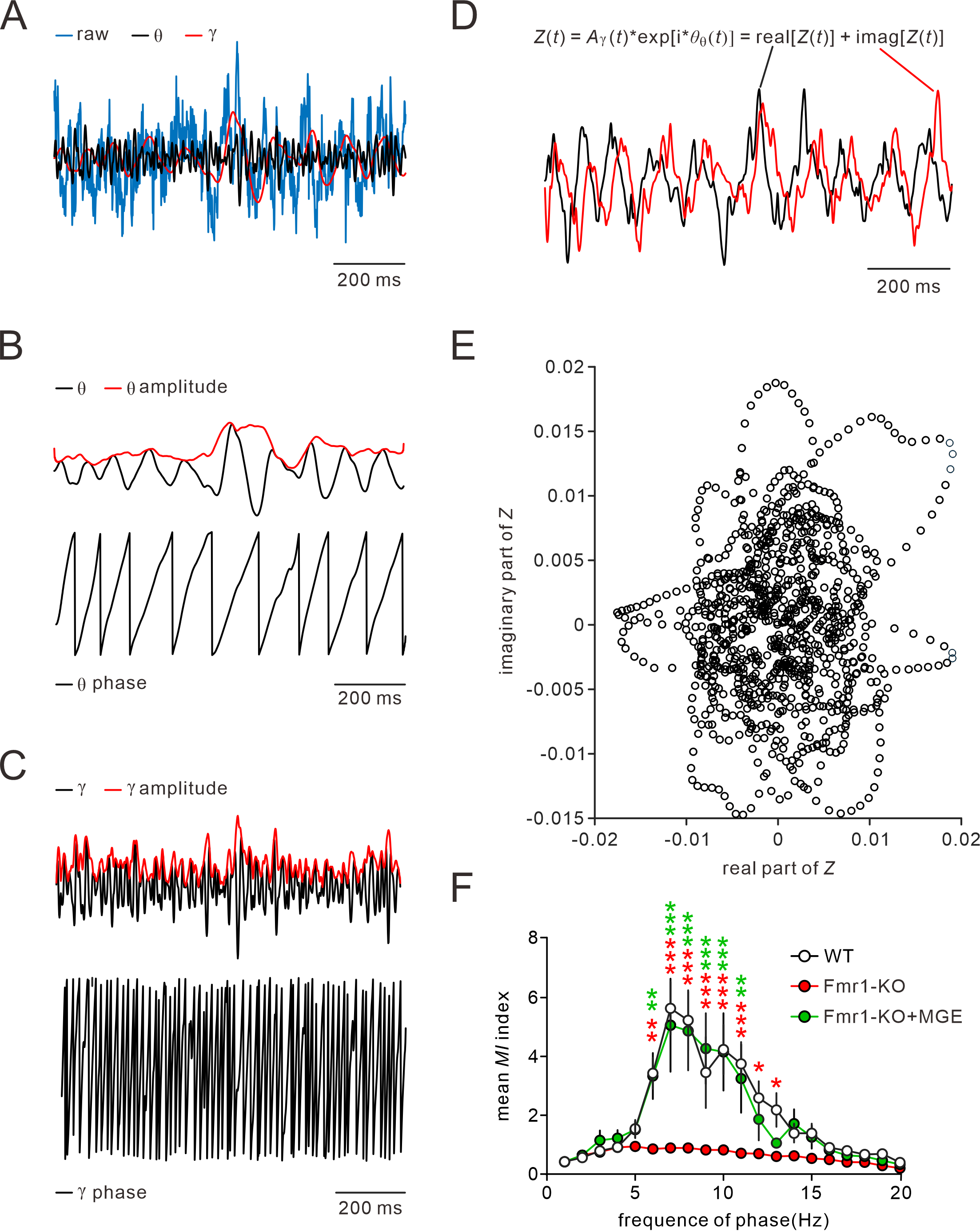
**

**Figure S15.** Correlation analysis of θ oscillation phase and γ oscillation amplitude. A) LFP signal unfiltered (blue), filtered in θ (4-12 Hz) range (black), and filtered in γ (20-100 Hz) range (red). B) Upper: time course of θ filtered trace from A (black) and θ analytic amplitude (red), also known as amplitude envelope. Lower: time course of θ analytic phase. C) Upper: time courses of γ filtered trace from panel A (black) and γ analytic amplitude (red). Lower: time course of γ analytic phase. D) Real (black) and imaginary (red) parts of analytic signal *Z*(*t*) formed by combining γ analytic amplitude time course with θ analytic phase time course. E) Values of *Z*(*t*) for all sample points examined. F) Mean *MI* values for γ analytic amplitude and analytic phases at 1-20 Hz. Red and green asterisks indicate significance levels of WT *vs* Fmr1-KO or Fmr1-KO *vs* Fmr1-KO+MGE. See Table S11 (Supporting Information) for statistics, including *n* values, *p* values, and specific statistical tests. ****p* < 0.001.

**
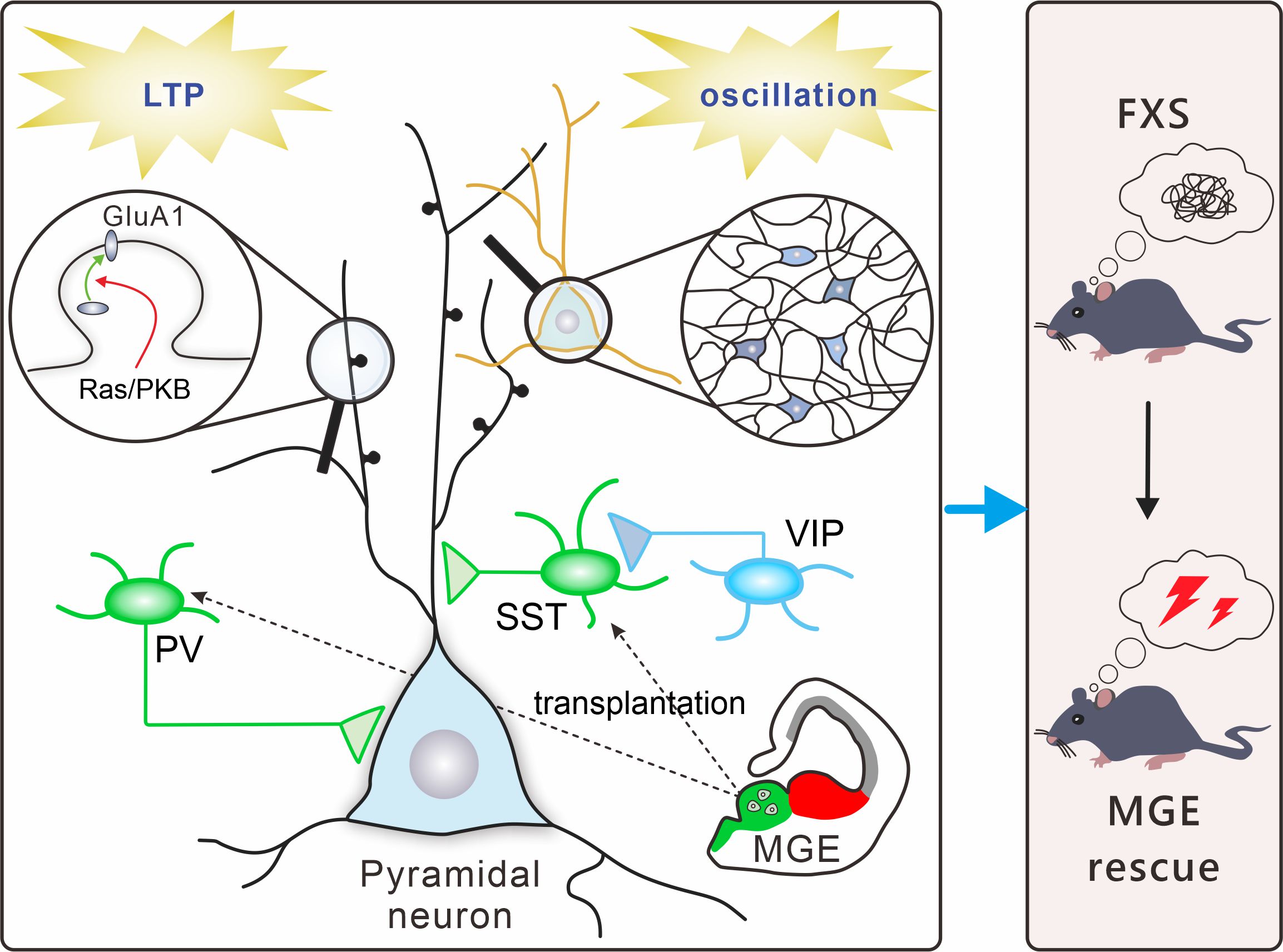
**

**Figure S16**. Summary of the present work. MGE cell transplantation can repair Ras-PKB signaling, AMPAR transport and synaptic plasticity in FXS mice; MGE cell transplantation can repair the damaged neuronal oscillation in FXS mice; and hippocampal transplantation of MGE cells rescues the cognitive disorders of FXS mice.

**Table S1**

**Statistics for Figure 1D – Numbers of GFP^+^ cells at 7 DAT, 14 DAT, 30 DAT and 60 DAT**

|  | **numbers of GFP^+^ cells (x10^3^)** | ***p* values** | **mouse number** |
| --- | --- | --- | --- |
| 7 DAT | 20.5 ± 0.8 | 0.78 (*vs* 14 DAT)  0.28 (*vs* 30 DAT)  0.16 (*vs* 60 DAT) | 5 |
| 14 DAT | 21.5 ± 0.7 | 0.80 (*vs* 7 DAT)  0.78 (*vs* 30 DAT)  0.57 (*vs* 60 DAT) | 5 |
| 30 DAT | 22.5 ± 0.6 | 0.28 (*vs* 7 DAT)  0.78 (*vs* 14 DAT)  0.98 (*vs* 60 DAT) | 5 |
| 60 DAT | 22.9 ± 0.6 | 0.16 (*vs* 7 DAT)  0.57 (*vs* 14 DAT)  0.98 (*vs* 30 DAT) | 5 |

Two-way ANOVA test. Mean ± SEM.

**Statistics for Figure 1E – Distribution of transplanted MGE cells at 60 DAT**

| **distance** | **percentage of GFP^+^ cells** | ***mouse number*** |
| --- | --- | --- |
| -900 μm | 0.9 ± 0.2 | 5 |
| -600 μm | 3.6 ± 0.6 | 5 |
| -300 μm | 17.5 ± 0.9 | 5 |
| 0 | 45.7 ± 0.5 | 5 |
| 300 μm | 32.0 ± 0.9 | 5 |
| 600 μm | 12.0 ± 0.6 | 5 |
| 900 μm | 8.8 ± 0.3 | 5 |
| 1200 μm | 1.0 ± 0.3 | 5 |
| 1500 μm | 0.6 ± 0.2 | 5 |

**Table S2**

**Statistics for Figure 3C – Parameters of uIPSCs**

|  | **latency (μs)** | **Τ1 (ms)** | **T2 (ms)** | ***n*** |
| --- | --- | --- | --- | --- |
| *In situ* IN | 704.1 ± 24.6 | 0.91 ± 0.12 | 6.03 ± 0.52 | 12 |
| Differentiated IN | 723.6 ± 14.3 | 0.99 ± 0.09 | 6.13 ± 0.69 | 12 |
| *p* values | 0.24 | 0.31 | 0.45 |  |

Unpaired *t* test. Mean ± SEM.

**Statistics for Figure 3F, I – Parameters of mEPSCs and mIPSCs**

| **mIPSC** | **frequency (Hz)** | **amplitude (pA)** | ***n*** |
| --- | --- | --- | --- |
| Fmr1-KO | 5.2 ± 0.3 | 50.9 ± 5.2 | 10 |
| Fmr1-KO+MGE | 6.7 ± 0.4 | 76.3 ± 9.6 | 10 |
| *p* values | 0.00068 | 0.0083 |  |
| **mEPSC** | **frequency (Hz)** | **amplitude (pA)** | ***n*** |
| Fmr1-KO | 2.3 ± 0.2 | 14.3 ± 1.1 | 10 |
| Fmr1-KO+MGE | 2.2 ± 0.2 | 14.4 ± 1.4 | 10 |
| *p* values | 0.41 | 0.47 |  |

Unpaired *t* test. Mean ± SEM.

**Table S3**

**Statistics for Figure 4A – Latency to escape to visible platform**

| **latency (s)** | **WT** | **Fmr1-KO** | **Fmr1-KO+MGE** | **Fmr1-KO+dMGE** |
| --- | --- | --- | --- | --- |
| day 1 | 8.7 ± 0.9 | 13.5 ± 1.3 | 11.4 ± 1.1 | 8.9 ± 0.7 |
| day 2 | 6.2 ± 0.3 | 7.1 ± 0.6 | 6.9 ± 0.4 | 6.2 ± 0.3 |
| *p* values | 0.42 (*vs* Fmr1-KO)  0.79 (*vs* Fmr1-KO+MGE)  0.99 (*vs* Fmr1-KO+dMGE) | 0.90 (*vs* Fmr1-KO+MGE)  0.50 (*vs* Fmr1-KO+dMGE) | 0.85 (*vs* Fmr1-KO+dMGE) |  |
|  | **WT** | **Fmr1-KO** | **Fmr1-KO+MGE** | **Fmr1-KO+dMGE** |
| speed (cm/s) | 23.0 ± 0.8 | 23.0 ± 0.6 | 23.7 ± 0.6 | 22.1 ± 0.4 |
| *p* values | 0.99 (*vs* Fmr1-KO)  0.90 (*vs* Fmr1-KO+MGE)  0.78 (*vs* Fmr1-KO+dMGE) | 0.87 (*vs* Fmr1-KO+MGE)  0.84 (*vs* Fmr1-KO+dMGE) | 0.39 (*vs* Fmr1-KO+dMGE) |  |
| *n* | 5 | 6 | 5 | 6 |

Two-way ANOVA test. Mean ± SEM.

**Statistics for Figure 4B – Latency to escape to invisible platform**

| **Probe 1**  **latency (s)** | **WT** | **Fmr1-KO** | **Fmr1-KO+MGE** | **Fmr1-KO+dMGE** |
| --- | --- | --- | --- | --- |
| day 1 | 50.1 ± 1.5 | 52.2 ± 1.2 | 51.3 ± 1.1 | 49.1 ± 1.2 |
| *p* values | 0.25 (*vs* Fmr1-KO)  0.48 (*vs* Fmr1-KO+MGE)  0.61 (*vs* Fmr1-KO+dMGE) | 0.62 (*vs* Fmr1-KO+MGE)  0.12 (*vs* Fmr1-KO+dMGE) | 0.25 (*vs* Fmr1-KO+dMGE) |  |
| day 2 | 40.3 ± 0.6 | 42.5 ± 0.8 | 41.5 ± 0.5 | 43.1 ± 1.2 |
| *p* values | 0.062 (*vs* Fmr1-KO)  0.29 (*vs* Fmr1-KO+MGE)  0.059 (*vs* Fmr1-KO+dMGE) | 0.36 (*vs* Fmr1-KO+MGE)  0.58 (*vs* Fmr1-KO+dMGE) | 0.14 (*vs* Fmr1-KO+dMGE) |  |
| day 3 | 15.2 ± 0.9 | 30.1 ±1.4 | 14.4 ± 0.9 | 30.3 ± 1.6 |
| *p* values | < 0.001 (*vs* Fmr1-KO)  0.63 (*vs* Fmr1-KO+MGE)  < 0.001 (*vs* Fmr1-KO+dMGE) | < 0.001 (*vs* Fmr1-KO+MGE)  0.92 (*vs* Fmr1-KO+dMGE) | < 0.001 (*vs* Fmr1-KO+dMGE) |  |
| day 4 | 10.1 ± 0.70 | 26.1 ± 0.91 | 11.2 ± 0.69 | 27.5 ± 1.8 |
| *p* values | < 0.001 (*vs* Fmr1-KO)  0.49 (*vs* Fmr1-KO+MGE)  < 0.001 (*vs* Fmr1-KO+dMGE) | < 0.001 (*vs* Fmr1-KO+MGE)  0.38 (*vs* Fmr1-KO+dMGE) | < 0.001 (*vs* Fmr1-KO+dMGE) |  |
| *n* | 6 | 5 | 6 | 5 |
| **Probe 2**  **latency (s)** | **WT** | **Fmr1-KO** | **Fmr1-KO+MGE** | **Fmr1-KO+dMGE** |
| day 1 | 55.1 ± 1.1 | 55.3 ± 1.5 | 54.5 ± 1.0 | 56.1 ± 1.5 |
| *p* values | 0.92 (*vs* Fmr1-KO)  0.73 (*vs* Fmr1-KO+MGE)  0.59 (*vs* Fmr1-KO+dMGE) | 0.66 (*vs* Fmr1-KO+MGE)  0.68 (*vs* Fmr1-KO+dMGE) | 0.39 (*vs* Fmr1-KO+dMGE) |  |
| day 2 | 44.3 ± 1.2 | 46.1 ± 2.1 | 43.3 ± 1.1 | 50.3 ± 2.1 |
| *p* values | 0.43 (*vs* Fmr1-KO)  0.67 (*vs* Fmr1-KO+MGE)  0.053 (*vs* Fmr1-KO+dMGE) | 0.24 (*vs* Fmr1-KO+MGE)  0.085 (*vs* Fmr1-KO+dMGE) | 0.006 (*vs* Fmr1-KO+dMGE) |  |
| day 3 | 20.8 ± 1.8 | 35.3 ± 1.6 | 21.2 ± 1.1 | 35.1 ± 1.9 |
| *p* values | < 0.001 (*vs* Fmr1-KO)  0.83 (*vs* Fmr1-KO+MGE)  < 0.001 (*vs* Fmr1-KO+dMGE) | < 0.001 (*vs* Fmr1-KO+MGE)  0.96 (*vs* Fmr1-KO+dMGE) | < 0.001 (*vs* Fmr1-KO+dMGE) |  |
| day 4 | 9.9 ± 1.1 | 30.1 ± 1.5 | 10.1 ± 1.0 | 31.1 ± 1.4 |
| *p* values | < 0.001 (*vs* Fmr1-KO)  0.89 (*vs* Fmr1-KO+MGE)  < 0.001 (*vs* Fmr1-KO+dMGE) | < 0.001 (*vs* Fmr1-KO+MGE)  0.59 (*vs* Fmr1-KO+dMGE) | < 0.001 (*vs* Fmr1-KO+dMGE) |  |
| *n* | 6 | 5 | 6 | 5 |

Repeated measures ANOVA. Mean ± SEM.

**Statistics for Figure 4D – Platform crossings**

| **Probe 1** | **WT** | **Fmr1-KO** | **Fmr1-KO+MGE** | **Fmr1-KO+dMGE** |
| --- | --- | --- | --- | --- |
| crossings | 10.2 ± 0.5 | 4.4 ± 0.5 | 10.0 ± 0.6 | 5.0 ± 0.5 |
| *p* values | < 0.001 (*vs* Fmr1-KO)  0.99 (*vs* Fmr1-KO+MGE)  < 0.001 (*vs* Fmr1-KO+dMGE) | < 0.001 (*vs* Fmr1-KO+MGE)  0.89 (*vs* Fmr1-KO+dMGE) | < 0.001 (*vs* Fmr1-KO+dMGE) |  |
| *n* | 6 | 5 | 6 | 5 |
| **Probe 2** | **WT** | **Fmr1-KO** | **Fmr1-KO+MGE** | **Fmr1-KO+dMGE** |
| crossings | 11.2 ± 0.6 | 5.0 ± 0.4 | 10.3 ± 0.5 | 5.2 ± 0.5 |
| *p* values | < 0.001 (*vs* Fmr1-KO)  0.65 (*vs* Fmr1-KO+MGE)  < 0.001 (*vs* Fmr1-KO+dMGE) | < 0.001 (*vs* Fmr1-KO+MGE)  0.99 (*vs* Fmr1-KO+dMGE) | < 0.001 (*vs* Fmr1-KO+dMGE) |  |
| *n* | 6 | 5 | 6 | 5 |

Two-way ANOVA test. Mean ± SEM.

**Statistics for Figure 4E – Preference for target quadrant**

| **Quadrant**  **Percentage** | **target** | **left** | **right** | **opposite** | ***n*** |
| --- | --- | --- | --- | --- | --- |
| WT | 53.5 ± 2.2 | 20.5 ± 1.5 | 10.8 ± 1.1 | 15.2 ± 1.2 | 6 |
| Fmr1-KO | 39.3 ± 2.2 | 26.2 ± 1.4 | 17.1 ± 1.1 | 17.4 ± 1.5 | 5 |
| Fmr1-KO+MGE | 48.1 ± 2.6 | 24.8 ± 1.3 | 9.9 ± 1.0 | 17.2 ± 1.0 | 6 |
| Fmr1-KO+dMGE | 31.7 ± 2.7 | 23.5 ± 1.4 | 24.1 ± 1.4 | 20.7 ± 1.2 | 5 |

Mean ± SEM.

**Statistics for Figure 4F – Time spent with objects**

| **%** | **FPO** | **NPO** | ***p* values** | ***n*** |
| --- | --- | --- | --- | --- |
| WT | 27.6 ± 1.4 | 72.4 ± 1.3 | < 0.001 | 5 |
| Fmr1-KO | 60.2 ± 1.2 | 39.8 ± 1.2 | 0.002 | 8 |
| Fmr1-KO+MGE | 30.2 ± 1.1 | 69.8 ± 1.3 | < 0.001 | 7 |
| Fmr1-KO+dMGE | 57.1 ± 0.9 | 42.9 ± 0.8 | < 0.001 | 5 |

Unpaired *t* test. Mean ± SEM.

**Statistics for Figure 4G – Preference to NPO**

|  | **WT** | **Fmr1-KO** | **Fmr1-KO+MGE** | **Fmr1-KO+dMGE** |
| --- | --- | --- | --- | --- |
| Preference | 44.8 ± 2.0 | -20.4 ± 1.7 | 39.7 ± 1.5 | -14.2 ± 1.0 |
| *p* values | < 0.001 (*vs* Fmr1-KO)  0.95 (*vs* Fmr1-KO+MGE)  < 0.001 (*vs* Fmr1-KO+dMGE) | < 0.001 (*vs* Fmr1-KO+MGE)  0.91 (*vs* Fmr1-KO+dMGE) | < 0.001 (*vs* Fmr1-KO+dMGE) |  |
| *n* | 5 | 8 | 7 | 5 |

One-way ANOVA test. Mean ± SEM.

**Statistics for Figure 4G – Time spent with objects**

| **%** | **FOA** | **NOA** | ***p* values** | ***n*** |
| --- | --- | --- | --- | --- |
| WT | 38.3 ± 1.6 | 61.7 ± 1.8 | < 0.001 | 5 |
| Fmr1-KO | 63.2 ± 1.2 | 36.8 ± 1.1 | < 0.001 | 8 |
| Fmr1-KO+MGE | 39.6 ± 0.9 | 60.4 ± 1.2 | < 0.001 | 7 |
| Fmr1-KO+dMGE | 56.7 ± 1.3 | 43.3 ± 1.2 | 0.008 | 5 |

Unpaired *t* test. Mean ± SEM.

**Statistics for Figure 4G – Preference to NOA**

|  | **WT** | **Fmr1-KO** | **Fmr1-KO+MGE** | **Fmr1-KO+dMGE** |
| --- | --- | --- | --- | --- |
| Preference | 23.5 ± 2.3 | -26.4 ± 1.7 | 20.7 ± 1.3 | -13.4 ± 2.5 |
| *p* values | < 0.001 (*vs* Fmr1-KO)  0.99 (*vs* Fmr1-KO+MGE)  0.01 (*vs* Fmr1-KO+dMGE) | < 0.001 (*vs* Fmr1-KO+MGE)  0.53 (*vs* Fmr1-KO+dMGE) | 0.01 (*vs* Fmr1-KO+dMGE) |  |
| *n* | 5 | 8 | 7 | 5 |

One-way ANOVA test. Mean ± SEM.

**Statistics for Figure 4H – Freezing time**

| **Freezing time %** | **Train** | **Context** | **Tone** | ***n*** |
| --- | --- | --- | --- | --- |
| WT | 34.3 ± 0.9 | 68.0 ± 1.2 | 43.9 ± 1.5 | 10 |
| Fmr1-KO | 38.9 ± 0.8 | 41.9 ± 1.3 | 29.4 ± 1.1 | 14 |
| Fmr1-KO+MGE | 36.1 ± 1.0 | 63.4 ± 1.2 | 31.9 ± 1.3 | 8 |
| Fmr1-KO+dMGE | 35.0 ± 1.1 | 38.2 ± 1.5 | 34.0 ± 1.4 | 5 |
| *p* values | 0.46 (WT *vs* Fmr1-KO)  0.96 (WT *vs* Fmr1-KO+MGE)  0.99 (WT *vs* Fmr1-KO+dMGE)  0.83 (Fmr1-KO *vs* Fmr1-KO+MGE)  0.75 (Fmr1-KO *vs* Fmr1-KO+dMGE)  0.99 (Fmr1-KO+MGE *vs* Fmr1-KO+dMGE) | < 0.001 (WT *vs* Fmr1-KO)  0.55 (WT *vs* Fmr1-KO+MGE)  0.002 (WT *vs* Fmr1-KO+dMGE)  0.005 (Fmr1-KO *vs* Fmr1-KO+MGE)  0.66 (Fmr1-KO *vs* Fmr1-KO+dMGE)  0.009(Fmr1-KO+MGE *vs* Fmr1-KO+dMGE) | 0.024 (WT *vs* Fmr1-KO)  0.038 (WT *vs* Fmr1-KO+MGE)  0.049 (WT *vs* Fmr1-KO+dMGE)  0.71 (Fmr1-KO *vs* Fmr1-KO+MGE)  0.56 (Fmr1-KO *vs* Fmr1-KO+dMGE)  0.80 (Fmr1-KO+MGE *vs* Fmr1-KO+dMGE) |  |

One-way ANOVA test. Mean ± SEM.

**Table S4**

**Statistics for Fig. S5B-D – Social ability in three chamber test**

| **Time %** | **WT** | **Fmr1-KO** | **Fmr1-KO+MGE** | **Fmr1-KO+dMGE** |
| --- | --- | --- | --- | --- |
| S1 | 58.5 ± 1.4 | 59.5 ± 1.4 | 55.8 ± 1.2 | 48.9 ± 1.3 |
| mid | 13.3 ± 0.8 | 12.4 ± 0.8 | 11.3 ± 0.7 | 16.6 ± 0.9 |
| obj | 28.1 ± 1.3 | 28.1 ± 1.4 | 33.0 ± 1.2 | 34.6 ± 0.9 |
| *n* | 7 | 9 | 9 | 5 |

Mean ± SEM.

**Statistics for Fig. S5F-H – Social novelty in three chamber test**

| **Time %** | **WT** | **Fmr1-KO** | **Fmr1-KO+MGE** | **Fmr1-KO+dMGE** |
| --- | --- | --- | --- | --- |
| S1 | 32.8 ± 1.3 | 48.8 ± 1.3 | 27.0 ± 1.1 | 54.7 ± 1.9 |
| mid | 13.7 ± 1.0 | 15.3 ± 0.9 | 15.0 ± 1.1 | 18.2 ± 1.2 |
| S2 | 53.5 ± 1.4 | 36.0 ± 1.1 | 58.1 ± 1.2 | 27.0 ± 1.5 |
| *n* | 7 | 9 | 9 | 5 |

Mean ± SEM.

**Statistics for Figure S5 – Parameters of three chamber test**

| **Freezing time %** | **S1 preference** | **S1 sniffing time** | **S2 preference** | **S2 sniffing time** |
| --- | --- | --- | --- | --- |
| WT | 34.5 ± 17.2 | 248.5 ± 18.2 | 38.2 ± 10.2 | 143.9 ± 12.5 |
| Fmr1-KO | 36.8 ± 23.5 | 236.0 ± 10.5 | -12.1 ± 8.5 | 69.0 ± 7.0 |
| Fmr1-KO+MGE | 26.5 ± 15.2 | 248.0 ± 10.0 | 32.5 ± 7.6 | 132.8 ± 10.0 |
| Fmr1-KO+dMGE | 16.8 ± 10.0 | 220.0 ± 4.2 | -32.4 ± 12.6 | 83.5 ± 4.5 |
| *p* values | 0.99 (WT *vs* Fmr1-KO)  0.80 (WT *vs* Fmr1-KO+MGE)  0.65 (WT *vs* Fmr1-KO+dMGE)  0.70 (Fmr1-KO *vs* Fmr1-KO+MGE)  0.56 (Fmr1-KO *vs* Fmr1-KO+dMGE)  0.98 (Fmr1-KO+MGE *vs* Fmr1-KO+dMGE) | 0.77 (WT *vs* Fmr1-KO)  0.99 (WT *vs* Fmr1-KO+MGE)  0.48 (WT *vs* Fmr1-KO+dMGE)  0.83 (Fmr1-KO *vs* Fmr1-KO+MGE)  0.92 (Fmr1-KO *vs* Fmr1-KO+dMGE)  0.53 (Fmr1-KO+MGE *vs* Fmr1-KO+dMGE) | 0.007 (WT *vs* Fmr1-KO)  0.73 (WT *vs* Fmr1-KO+MGE)  0.005 (WT *vs* Fmr1-KO+dMGE)  0.001 (Fmr1-KO *vs* Fmr1-KO+MGE)  0.57 (Fmr1-KO *vs* Fmr1-KO+dMGE)  < 0.001 (Fmr1-KO+MGE *vs* Fmr1-KO+dMGE) | < 0.001 (WT *vs* Fmr1-KO)  0.64 (WT *vs* Fmr1-KO+MGE)  0.003 (WT *vs* Fmr1-KO+dMGE)  < 0.001 (Fmr1-KO *vs* Fmr1-KO+MGE)  0.68 (Fmr1-KO *vs* Fmr1-KO+dMGE)  0.005 (Fmr1-KO+MGE *vs* Fmr1-KO+dMGE) |

One-way ANOVA test. Mean ± SEM.

**Table S5**

**Statistics for Figure S6B – Parameters of OFT**

|  | **inner zone (s)** | **outer zone (s)** | **distance (m)** | ***n*** |
| --- | --- | --- | --- | --- |
| WT | 138.4 ± 2.7 | 461.6 ± 2.7 | 34.9 ± 1.4 | 7 |
| Fmr1-KO | 73.4 ± 1.7 | 526.6 ± 1.7 | 32.3 ± 0.9 | 5 |
| Fmr1-KO+MGE | 163.6 ± 1.2 | 436.4 ± 1.2 | 40.6 ± 1.7 | 7 |
| Fmr1-KO+dMGE | 75.5 ± 2.5 | 524.6 ± 2.5 | 53.6 ± 1.6 | 5 |
| *p* values | 0.007 (WT *vs* Fmr1-KO)  0.42 (WT *vs* Fmr1-KO+MGE)  0.01 (WT *vs* Fmr1-KO+dMGE)  < 0.001 (Fmr1-KO *vs* Fmr1-KO+MGE)  0.99 (Fmr1-KO *vs* Fmr1-KO+dMGE)  < 0.001 (Fmr1-KO+MGE *vs* Fmr1-KO+dMGE) | 0.007 (WT *vs* Fmr1-KO)  0.42 (WT *vs* Fmr1-KO+MGE)  0.01 (WT *vs* Fmr1-KO+dMGE)  < 0.001 (Fmr1-KO *vs* Fmr1-KO+MGE)  0.99 (Fmr1-KO *vs* Fmr1-KO+dMGE)  < 0.001 (Fmr1-KO+MGE *vs* Fmr1-KO+dMGE) | 0.99 (WT *vs* Fmr1-KO)  0.87 (WT *vs* Fmr1-KO+MGE)  0.14 (WT *vs* Fmr1-KO+dMGE)  0.75 (Fmr1-KO *vs* Fmr1-KO+MGE)  0.12 (Fmr1-KO *vs* Fmr1-KO+dMGE)  0.42 (Fmr1-KO+MGE *vs* Fmr1-KO+dMGE) |  |

One-way ANOVA test. Mean ± SEM.

**Statistics for Figure S6D,F,H – Parameters of repetitive behaviors**

|  | **WT** | **Fmr1-KO** | **Fmr1-KO+MGE** | **Fmr1-KO+dMGE** |
| --- | --- | --- | --- | --- |
| Marbles buried (%) | 8.1 ± 0.8 | 17.8 ± 0.7 | 10.5 ± 0.5 | 21.9 ± 0.8 |
| *p* values | < 0.001 (*vs* Fmr1-KO)  0.76 (*vs* Fmr1-KO+MGE)  < 0.001 (*vs* Fmr1-KO+dMGE) | 0.005 (*vs* Fmr1-KO+MGE)  0.32 (*vs* Fmr1-KO+dMGE) | < 0.001 (*vs* Fmr1-KO+dMGE) |  |
| *n* | 7 | 11 | 6 | 5 |
| number of repeats | 2.7 ± 1.3 | 3.8 ± 0.5 | 2.3 ± 0.5 | 3.2 ± 0.6 |
| *p* values | 0.53 (*vs* Fmr1-KO)  0.97 (*vs* Fmr1-KO+MGE)  0.93 (*vs* Fmr1-KO+dMGE) | 0.24 (*vs* Fmr1-KO+MGE)  0.91 (*vs* Fmr1-KO+dMGE) | 0.71 (*vs* Fmr1-KO+dMGE) |  |
| *n* | 6 | 8 | 7 | 5 |
| interrupted bouts | 4.6 ± 1.3 | 4.8 ± 0.5 | 4.9 ± 0.5 | 4.2 ± 0.6 |
| *p* values | 0.98 (*vs* Fmr1-KO)  0.98 (*vs* Fmr1-KO+MGE)  0.97 (*vs* Fmr1-KO+dMGE) | 0.99 (*vs* Fmr1-KO+MGE)  0.85 (*vs* Fmr1-KO+dMGE) | 0.86 (*vs* Fmr1-KO+dMGE) |  |
| time on self-grooming (s) | 148.3 ± 2.8 | 140.7 ± 2.0 | 149.9 ± 2.3 | 129.8 ± 3.3 |
| *p* values | 0.98 (*vs* Fmr1-KO)  0.99 (*vs* Fmr1-KO+MGE)  0.88 (*vs* Fmr1-KO+dMGE) | 0.97 (*vs* Fmr1-KO+MGE)  0.96 (*vs* Fmr1-KO+dMGE) | 0.85 (*vs* Fmr1-KO+dMGE) |  |
| *n* | 7 | 11 | 7 | 5 |

One-way ANOVA test. Mean ± SEM.

**Statistics for Figure S6I – Parameters of rotarod**

| **Latency to fall (s)** | **WT** | **Fmr1-KO** | **Fmr1-KO+MGE** | ***p* values** |
| --- | --- | --- | --- | --- |
| session 1 | 74.6 ± 13.8 | 51.0 ± 11.0 | 45.6 ± 7.2 | 0.127 (WT *vs* Fmr1-KO)  0.090 (WT *vs* Fmr1-KO+MGE)  0.74 (Fmr1-KO *vs* Fmr1-KO+dMGE) |
| session 2 | 91.3 ± 21.4 | 55.9 ± 11.0 | 54.4 ± 7.9 | 0.092 (WT *vs* Fmr1-KO)  0.11 (WT *vs* Fmr1-KO+MGE)  0.95 (Fmr1-KO *vs* Fmr1-KO+dMGE) |
| session 3 | 109.7 ± 23.4 | 72.6 ± 15.1 | 60.2 ± 13.4 | 0.13 (WT *vs* Fmr1-KO)  0.072 (WT *vs* Fmr1-KO+MGE)  0.64 (Fmr1-KO *vs* Fmr1-KO+dMGE) |
| session 4 | 105.7 ± 18.1 | 74.7 ± 15.1 | 59.6 ± 4.7 | 0.12 (WT *vs* Fmr1-KO)  0.042 (WT *vs* Fmr1-KO+MGE)  0.48 (Fmr1-KO *vs* Fmr1-KO+dMGE) |
| session 5 | 109.4 ± 19.5 | 67.9 ± 11.6 | 68.0 ± 8.4 | 0.041 (WT *vs* Fmr1-KO)  0.048 (WT *vs* Fmr1-KO+MGE)  0.99 (Fmr1-KO *vs* Fmr1-KO+dMGE) |
| session 6 | 135.0 ± 24.0 | 80.0 ± 14.4 | 82.4 ± 10.7 | 0.030 (WT *vs* Fmr1-KO)  0.045 (WT *vs* Fmr1-KO+MGE)  0.93 (Fmr1-KO *vs* Fmr1-KO+dMGE) |
| session 7 | 130.9 ± 21.2 | 88.4 ± 13.7 | 78.0 ± 13.6 | 0.046 (WT *vs* Fmr1-KO)  0.039 (WT *vs* Fmr1-KO+MGE)  0.66 (Fmr1-KO *vs* Fmr1-KO+dMGE) |
| session 8 | 132.3 ± 19.9 | 78.7 ± 16.6 | 79.0 ± 17.4 | 0.031 (WT *vs* Fmr1-KO)  0.048 (WT *vs* Fmr1-KO+MGE)  0.99 (Fmr1-KO *vs* Fmr1-KO+dMGE) |
| *n* | 7 | 7 | 5 |  |

Repeated measures ANOVA. Mean ± SEM.

**Table S6**

**Statistics for Figure 5B – Fiber volley**

| **mV** | **25 μA** | **45 μA** | **85 μA** | **160 μA** | ***n*** |
| --- | --- | --- | --- | --- | --- |
| WT | 0.039 ± 0.006 | 0.20 ± 0.06 | 0.50 ± 0.08 | 0.82 ± 0.06 | 5 |
| Fmr1-KO | 0.044 ± 0.006 | 0.17 ± 0.03 | 0.43 ± 0.04 | 0.89 ± 0.02 | 5 |
| Fmr1-KO+MGE | 0.043 ± 0.007 | 0.18 ± 0.03 | 0.45 ± 0.07 | 0.84 ± 0.05 | 5 |
| *p* values | 0.99 WT *vs* Fmr1-KO  0.95 WT *vs* Fmr1-KO+MGE  0.98 Fmr1-KO *vs* Fmr1-KO+MGE | 0.65 WT *vs* Fmr1-KO  0.68 WT *vs* Fmr1-KO+MGE  0.96 Fmr1-KO *vs* Fmr1-KO+MGE | 0.25 WT *vs* Fmr1-KO  0.39 WT *vs* Fmr1-KO+MGE  0.77 Fmr1-KO *vs* Fmr1-KO+MGE | 0.20 WT *vs* Fmr1-KO  0.68 WT *vs* Fmr1-KO+MGE  0.38 Fmr1-KO *vs* Fmr1-KO+MGE |  |

two-way ANOVA. Mean ± SEM.

**Statistics for Figure 5E – LTP amplitude**

| **%** | **WT** | **Fmr1-KO** | **Fmr1-KO+MGE** | ***n*** |
| --- | --- | --- | --- | --- |
| 1 | 100 ± 1.8 | 100 ± 1.5 | 100 ± 1.6 | 5 |
| 2 | 199.9 ± 14.2 | 145.5 ± 9.0 | 189.2 ± 21.8 | 5 |
| *p* values | < 0.001 | 0.0013 | 0.00037 |  |

two-way ANOVA. Mean ± SEM.

**Table S7**

**Statistics for Figure 6I – Change of activated Ras**

| % | **WT** | **Fmr1-KO** | **Fmr1-KO+MGE** | **Fmr1-KO+**  **dMGE** |
| --- | --- | --- | --- | --- |
| Ras-GTP | 100.0 ± 4.8 | 149.1 ± 4.8 | 100.7 ± 8.1 | 150.5 ± 10.2 |
| *p* values | < 0.001 (*vs* Fmr1-KO)  0.001 (*vs* Fmr1-KO+MGE)  < 0.001 (*vs* Fmr1-KO+dMGE) | < 0.001 (*vs* Fmr1-KO+MGE)  0.001 (*vs* Fmr1-KO+dMGE) | < 0.001 (*vs* Fmr1-KO+dMGE) |  |
| *n* | 5 | 5 | 5 | 5 |

One-way ANOVA test. Mean ± SEM.

**Statistics for Figure 6J – Change of phospho-PKB**

| % | **WT** | **Fmr1-KO** | **Fmr1-KO+MGE** | **Fmr1-KO+dMGE** |
| --- | --- | --- | --- | --- |
| pPKB | 100.0 ± 7.4 | 79.9 ± 7.9 | 105.3 ± 9.6 | 70.4 ± 6.2 |
| *p* values | 0.0045 (*vs* Fmr1-KO)  0.001 (*vs* Fmr1-KO+MGE)  < 0.001 (*vs* Fmr1-KO+dMGE) | 0.0012 (*vs* Fmr1-KO+MGE)  0.001 (*vs* Fmr1-KO+dMGE) | 0.0064 (*vs* Fmr1-KO+dMGE) |  |
| *n* | 5 | 5 | 5 | 5 |

One-way ANOVA test. Mean ± SEM.

**Table S8**

**Statistics for Figure 7B– Spike half-width and firing rate**

|  | **WT** | **Fmr1-KO** | **Fmr1-KO+MGE** |
| --- | --- | --- | --- |
| PNs firing rate | 4.7 ± 0.2 | 9.7 ± 0.2 | 4.3 ± 0.2 |
| *p* values | < 0.001 vs Fmr1-KO， | < 0.001 vs Fmr1-KO+MGE | 0.65 vs WT |
| INs firing rate | 26.1 ± 0.4 | 23.3 ± 0.5 | 28.2 ± 0.5 |
| *p* values | 0.039 vs Fmr1-KO | 0.0078 vs Fmr1-KO+MGE | 0.54 vs WT |
| PNs half-width | 0.2 ± 0.02 | 0.2 ± 0.03 | 0.2 ± 0.02 |
| *p* values | 0.87 vs Fmr1-KO | 0.98 vs Fmr1-KO+MGE | 0.76 vs WT |
| INs half-width | 0.2 ± 0.03 | 0.2 ± 0.04 | 0.25 ± 0.03 |
| *p* values | 0.99 vs Fmr1-KO | 0.75 vs Fmr1-KO+MGE | 0.71 vs WT |

One-way ANOVA test. Mean ± SEM.

**Table S9**

**Statistics for Figure 7C– Spike count correlations**

| **PN-PN**  **(s)** | **WT** | **Fmr1-KO** | **Fmr1-KO+**  **MGE** | ***p* values**  **(WT vs Fmr1-KO)** | ***p* values**  **(WT vs Fmr1-KO+**  **MGE)** | ***p* values**  **(Fmr1-KO**  **Vs Fmr1-KO+**  **MGE)** |
| --- | --- | --- | --- | --- | --- | --- |
| 0.1 | 0.04 ± 0.01 | 0.05 ± 0.01 | 0.04 ± 0.01 | < 0.001 | 0.392 | 0.002 |
| 0.2 | 0.05 ± 0.01 | 0.06 ± 0.01 | 0.05 ± 0.01 | < 0.001 | 0.99 | 0.001 |
| 0.3 | 0.05 ± 0.01 | 0.06 ± 0.01 | 0.05 ± 0.01 | 0.001 | 0.85 | < 0.001 |
| 0.4 | 0.05 ± 0.01 | 0.06 ± 0.01 | 0.05 ± 0.01 | 0.009 | 0.98 | 0.006 |
| 0.5 | 0.06 ± 0.01 | 0.07 ± 0.01 | 0.06 ± 0.01 | 0.60 | 0.46 | 0.092 |
| 0.6 | 0.07 ± 0.01 | 0.08 ± 0.01 | 0.07 ± 0.01 | 0.22 | 0.43 | 0.017 |
| 0.7 | 0.07 ± 0.01 | 0.08 ± 0.01 | 0.08 ± 0.01 | 0.49 | 0.49 | 0.99 |
| 0.8 | 0.08 ± 0.01 | 0.08 ± 0.01 | 0.08 ± 0.01 | 0.14 | 0.54 | 0.014 |
| 0.9 | 0.08 ± 0.01 | 0.09 ± 0.01 | 0.08 ± 0.01 | 0.027 | 0.55 | 0.22 |
| 1.0 | 0.09 ± 0.01 | 0.09 ± 0.01 | 0.09 ± 0.01 | 0.024 | 0.34 | 0.31 |

One-way ANOVA test. Mean ± SEM.

| **PN-IN**  **(s)** | **WT** | **Fmr1-KO** | **Fmr1-KO+**  **MGE** | ***p* values**  **(WT vs Fmr1-KO)** | ***p* values**  **(WT vs Fmr1-KO+**  **MGE)** | ***p* values**  **(Fmr1-KO**  **Vs Fmr1-KO+**  **MGE)** |
| --- | --- | --- | --- | --- | --- | --- |
| 0.1 | 0.05 ± 0.01 | 0.01 ± 0.01 | 0.04 ± 0.01 | < 0.001 | 0.043 | < 0.001 |
| 0.2 | 0.06 ± 0.01 | 0.01 ± 0.01 | 0.05 ± 0.01 | < 0.001 | 0.30 | < 0.001 |
| 0.3 | 0.06 ± 0.01 | 0.01 ± 0.01 | 0.06 ± 0.01 | < 0.001 | 0.99 | < 0.001 |
| 0.4 | 0.07 ± 0.01 | 0.01 ± 0.01 | 0.07 ± 0.01 | < 0.001 | 0.26 | < 0.001 |
| 0.5 | 0.08 ± 0.01 | 0.01 ± 0.01 | 0.08 ± 0.01 | < 0.001 | 0.001 | < 0.001 |
| 0.6 | 0.09 ± 0.01 | 0.02 ± 0.01 | 0.08 ± 0.01 | < 0.001 | 0.003 | < 0.001 |
| 0.7 | 0.09 ± 0.01 | 0.02 ± 0.004 | 0.09 ± 0.01 | < 0.001 | < 0.001 | < 0.001 |
| 0.8 | 0.09 ± 0.01 | 0.02 ± 0.01 | 0.09 ± 0.01 | < 0.001 | 0.006 | < 0.001 |
| 0.9 | 0.10 ± 0.01 | 0.02 ± 0.01 | 0.09 ± 0.01 | < 0.001 | 0.22 | < 0.001 |
| 1.0 | 0.10 ± 0.01 | 0.02 ± 0.01 | 0.09 ± 0.01 | < 0.001 | 0.82 | < 0.001 |

One-way ANOVA test. Mean ± SEM.

| **IN-IN**  **(s)** | **WT** | **Fmr1-KO** | **Fmr1-KO+**  **MGE** | ***p* values**  **(WT vs Fmr1-KO)** | ***p* values**  **(WT vs Fmr1-KO+**  **MGE)** | ***p* values**  **(Fmr1-KO**  **Vs Fmr1-KO+**  **MGE)** |
| --- | --- | --- | --- | --- | --- | --- |
| 0.1 | 0.3 ± 0.03 | 0.2 ± 0.018 | 0.25 ± 0.03 | < 0.001 | 0.13 | < 0.001 |
| 0.2 | 0.4 ± 0.02 | 0.2 ± 0.02 | 0.36 ± 0.03 | < 0.001 | 0.079 | < 0.001 |
| 0.3 | 0.5 ± 0.02 | 0.2 ± 0.03 | 0.46 ± 0.03 | < 0.001 | 0.96 | < 0.001 |
| 0.4 | 0.5 ± 0.03 | 0.2 ± 0.03 | 0.52 ± 0.03 | < 0.001 | 0.86 | < 0.001 |
| 0.5 | 0.5 ± 0.02 | 0.2 ± 0.03 | 0.55 ± 0.03 | < 0.001 | 0.99 | < 0.001 |
| 0.6 | 0.6 ± 0.02 | 0.2 ± 0.03 | 0.57 ± 0.02 | < 0.001 | 0.98 | < 0.001 |
| 0.7 | 0.6 ± 0.02 | 0.24 ± 0.03 | 0.58 ± 0.03 | < 0.001 | 0.91 | < 0.001 |
| 0.8 | 0.6 ± 0.02 | 0.24 ± 0.03 | 0.58 ± 0.03 | < 0.001 | 0.92 | < 0.001 |
| 0.9 | 0.6 ± 0.02 | 0.25 ± 0.03 | 0.59 ± 0.02 | < 0.001 | 0.89 | < 0.001 |
| 1.0 | 0.6 ± 0.02 | 0.25 ± 0.03 | 0.59 ± 0.02 | < 0.001 | 0.87 | < 0.001 |

One-way ANOVA test. Mean ± SEM.

**Table S10**

**Statistics for Figure 7F – Relative LFP powers**

| % | **WT** | **Fmr1-KO** | **Fmr1-KO+MGE** |
| --- | --- | --- | --- |
| θ | 38.2 ± 0.6 | 43.5 ± 0.6 | 35.2 ± 0.7 |
| *p* values | 0.0021 *vs* Fmr1-KO， | 0.00021 *vs* Fmr1-KO+MGE |  |
| low γ | 14.2 ± 0.6 | 7.9 ± 0.5 | 7.4 ± 0.5 |
| *p* values | 0.0074 *vs* Fmr1-KO | 0.038 *vs* Fmr1-KO+MGE |  |
| high γ | 9.8 ± 0.5 | 8.0 ± 0.5 | 6.5 ± 0.5 |
| *p* values | 0.63 *vs* Fmr1-KO | 0.22 *vs* Fmr1-KO+MGE |  |
| *n* | 13 | 10 | 15 |

One-way ANOVA test. Mean ± SEM.

**Table S11**

**Statistics for Figure S8F – *MI* index**

| **Frequency**  **(Hz)** | **WT** | **Fmr1-KO** | **Fmr1-KO+**  **MGE** | ***p* values**  **(WT vs Fmr1-KO)** | ***p* values**  **(WT vs Fmr1-KO+**  **MGE)** | ***p* values**  **(Fmr1-KO**  **Vs Fmr1-KO+**  **MGE)** |
| --- | --- | --- | --- | --- | --- | --- |
| 1 | 0.4 ± 0.05 | 0.4 ± 0.1 | 0.4 ± 0.04 | 0.99 | 0.99 | 0.99 |
| 2 | 0.6 ± 0.04 | 0.6 ± 0.1 | 0.6 ± 0.06 | 0.92 | 0.92 | 0.99 |
| 3 | 0.8 ± 0.05 | 0.7 ± 0.1 | 1.2 ± 0.4 | 0.95 | 0.63 | 0.59 |
| 4 | 0.9 ± 0.02 | 0.9 ± 0.16 | 1.2 ± 0.3 | 0.99 | 0.68 | 0.68 |
| 5 | 1.5 ± 0.3 | 0.9 ± 0.2 | 1.5 ± 0.3 | 0.42 | 0.97 | 0.45 |
| 6 | 3.4 ± 0.6 | 0.9 ± 0.03 | 3.3 ± 0.9 | 0.001 | 0.90 | 0.001 |
| 7 | 5.6 ± 1.0 | 0.9 ± 0.030 | 5.1 ± 1.7 | < 0.001 | 0.44 | < 0.001 |
| 8 | 5.2 ± 1.1 | 0.9 ± 0.08 | 4.9 ± 1.5 | < 0.001 | 0.63 | < 0.001 |
| 9 | 3.5 ± 1.3 | 0.8 ± 0.08 | 4.3 ± 1.3 | < 0.001 | 0.28 | < 0.001 |
| 10 | 4.2 ± 0.9 | 0.8 ± 0.06 | 4.2 ± 1.4 | < 0.001 | 0.92 | < 0.001 |
| 11 | 3.8 ± 0.8 | 0.7 ± 0.06 | 3. 3 ± 1.3 | < 0.001 | 0.50 | 0.001 |
| 12 | 2.6 ± 0.6 | 0.7 ± 0.07 | 1.9 ± 0.8 | 0.011 | 0.33 | 0.12 |
| 13 | 2.2 ± 0.6 | 0.6 ± 0.08 | 1.1 ± 0.1 | 0.035 | 0.13 | 0.54 |
| 14 | 1.4 ± 0.3 | 0.6 ± 0.09 | 1.7 ± 0.5 | 0.31 | 0.66 | 0.14 |
| 15 | 1.4 ± 0.4 | 0.5 ± 0.08 | 1.3 ± 0.4 | 0.25 | 0.86 | 0.33 |
| 16 | 0.9 ± 0.1 | 0.5 ± 0.06 | 0.8 ± 0.2 | 0.58 | 0.91 | 0.66 |
| 17 | 0.8 ± 0.07 | 0.4 ± 0.05 | 0.6 ± 0.1 | 0.60 | 0.83 | 0.76 |
| 18 | 0.7 ± 0.06 | 0.4 ± 0.04 | 0.6 ± 0.08 | 0.72 | 0.93 | 0.79 |
| 19 | 0.7 ± 0.2 | 0.3 ± 0.04 | 0.5 ± 007 | 0.59 | 0.76 | 0.82 |
| 20 | 0.4 ± 0.06 | 0.2 ± 0.03 | 0.3 ± 0.05 | 0.79 | 0.93 | 0.86 |

two-way ANOVA. Mean ± SEM.
